# Supplementary material for: Impacts of multisectoral cash plus programs after four years in an urban informal settlement: Adolescent Girls Initiative-Kenya (AGI-K) randomized trial
Source: PLoS One. 2022 Feb 7;17(2):e0262858. doi: 10.1371/journal.pone.0262858 (PMC8820646; doi:10.1371/journal.pone.0262858)
Supplement: S1 Table — (DOCX) [file pone.0262858.s001.docx]

**S1 Table: Key Indicators for AGI-K Primary and Secondary Outcomes**

| OUTCOME DOMAIN | INDICATOR 1 | INDICATOR 2 | INDICATOR 3 |
| --- | --- | --- | --- |
| PRIMARY OUTCOMES | |  |  |
| WELL-BEING | Age at first birth (+) | Age at first sex (+) | Age at marriage (+) |
| SECONDARY OUTCOMES | | | |
| VIOLENCE | Experience of gender-based violence (-) | Positive gender norms related to violence (+) |  |
| EDUCATION | Mean grade of schooling (+) | Rate of primary school completion (+) |  |
| HEALTH | Knowledge on sexual and reproductive health (+) | Decision-making skills (+) | Contraceptive use (+) |
| WEALTH | Knowledge on financial education (+) | Saving (+) | Participation in income generating activities (+) |
| Source: ISRCTN Trial registry (<http://www.isrctn.com/ISRCTN77455458>) first outlined in Institutional Review Board application to the Population Council and to the Kenyan AMREF Ethics and Scientific Review Committee, both approved in 2014, and reproduced in Austrian et al. 2016 (open access: <https://bmcpublichealth.biomedcentral.com/articles/10.1186/s12889-016-2888-1>). | | | |
