# Supplementary material for: Impacts of multisectoral cash plus programs after four years in an urban informal settlement: Adolescent Girls Initiative-Kenya (AGI-K) randomized trial
Source: PLoS One. 2022 Feb 7;17(2):e0262858. doi: 10.1371/journal.pone.0262858 (PMC8820646; doi:10.1371/journal.pone.0262858)
Supplement: S2 Table — (DOCX) [file pone.0262858.s002.docx]

**S2** **Table: Baseline means for full baseline sample, by study arm**

|  | (1) | | (2) | (3) | (4) | (5) |
| --- | --- | --- | --- | --- | --- | --- |
|  | V-Only | | VE | VEH | VEHW | Overall average |
| Background |  | |  |  |  |  |
| Age, mean (sd) | 12.6 (1.2) | | 12.5 (1.3) | 12.6 (1.2) | 12.5 (1.3) | 12.6 (1.3) |
| Cognitive score (0–16), mean (sd) [n=2,173] | 8.2 (3.0) | | 8.4 (3.0) | 8.3 (3.1) | 8.3 (3.2) | 8.3 (3.1) |
| Lives with both parents (=1), % [n=2,373] | 52.1 | | 55.0 | 50.0 | 52.6 | 52.4 |
| Mother completed primary school (=1), % [n=2,227] | 63.0 | | 62.8 | 62.7 | 64.55 | 63.3 |
| Father completed primary school (=1), % [n=1,962] | 76.3 | | 79.3 | 75.3 | 79.5 | 77.6 |
| Violence Prevention |  | |  |  |  |  |
| Experienced violence by a male in the past year (=1), % | 29.3 | | 29.9 | 31.4 | 33.1 | 30.9 |
| Positive gender attitudes score (0–4), mean (sd) | 3.6 (0.7) | | 3.6 (0.7) | 3.6 (0.7) | 3.6 (0.7) | 3.6 (0.7) |
| Education |  | |  |  |  |  |
| Grade attainment, mean (sd) | 5.7 (1.4) | | 5.7 (1.3) | 5.7 (1.4) | 5.7 (1.3) | 5.7 (1.4) |
| Primary school complete (=1), % | 8.0 | | 5.9 | 6.7 | 6.1 | 6.7 |
| Enrolled in current school year (=1), % | 99.3 | | 99.2 | 98.4 | 99.2 | 99.0 |
| Literate in Swahili and English (=1), % [n=2,373] | 91.9 | | 93.4 | 92.9 | 93.4 | 92.9 |
| Health |  | |  |  |  |  |
| Knows most fertile period during menstrual cycle (=1), % | 8.4 | | 8.3 | 7.1 | 6.1 | 7.4 |
| General self-efficacy score (0–6), mean (sd) | 3.8 (1.7) | | 4.0 (1.6) | 3.9 (1.6) | 4.0 (1.6) | 3.9 (1.6) |
| Wealth Creation |  | |  |  |  |  |
| Financial literacy score (0–10), mean (sd) | 5.7 (1.9) | | 5.6 (1.9) | 5.6 (1.9) | 5.8 (1.9) | 5.7 (1.9) |
| Saved money in the past six months (=1), % | 27.1 | | 25.2 | 26.4 | 28.4 | 26.8 |
| Worked for income in the last year (=1), % | 10.9 | | 12.0 | 11.7 | 10.0 | 11.1 |
| Reproductive health |  | |  |  |  |  |
| Ever had sex (=1), % | 1.7 | | 1.2 | 1.1 | 1.7 | 1.4 |
| Ever pregnant (=1), % | 0.4 | | 0.0 | 0.0 | 0.0 | 0.1 |
| Ever given birth (=1), % | 0.4 | | 0.0 | 0.0 | 0.0 | 0.1 |
| Household-level |  | |  |  |  |  |
| Household expects girl to complete secondary (=1), % [n=2,358] | | 99.8 | 99.7 | 99.7 | 99.8 | 99.7 |
| Household wealth quintile (1–5), mean (sd) [n=2,374] | 3.1 (1.4) | | 3.0 (1.4) | 3.0 (1.4) | 3.0 (1.4) | 3.0 (1.4) |
| Sample by arm when n=2,390 | 597 | | 592 | 609 | 592 | 2,390 |

Notes: N = 2,390 unless otherwise indicated. Sample sizes are larger for household-level variables because for some observations the household but not individual survey was completed. Final column is overall average. As recommended in CONSORT guidelines for randomized trials (Moher et al. 2010) we did not conduct statistical tests comparing treatment arms at baseline (although in the text we present such tests for the endline sample after attrition).

Reference:

Moher D, Hopewell S, Schulz KF, Montori V, Gøtzsche PC, Devereaux PJ, Elbourne D, Egger M, Altman DG. 2010. CONSORT 2010 explanation and elaboration: updated guidelines for reporting parallel group randomised trials. *Journal of Clinical Epidemiology* 63: e1–e3
