# Supplementary material for: Impacts of multisectoral cash plus programs after four years in an urban informal settlement: Adolescent Girls Initiative-Kenya (AGI-K) randomized trial
Source: PLoS One. 2022 Feb 7;17(2):e0262858. doi: 10.1371/journal.pone.0262858 (PMC8820646; doi:10.1371/journal.pone.0262858)
Supplement: S3 Table — (DOCX) [file pone.0262858.s003.docx]

**S3 Table: Baseline correlates of endline survey response, by study arm**

| Dependent variable: Interviewed at endline (=1) | | (1) | (2a) | (2b) | (2c) | (2d) |
| --- | --- | --- | --- | --- | --- | --- |
|  |  | | X | Study arm 2*X | Study arm 3*X | Study arm 4*X |
| Study arms: V-only (ref) |  | |  |  |  |  |
| VE (=1) | 0.083*** | | -0.139 |  |  |  |
|  | (0.021) | | (0.187) |  |  |  |
| VEH (=1) | 0.068*** | | -0.075 |  |  |  |
|  | (0.021) | | (0.170) |  |  |  |
| VEHW (=1) | 0.091*** | | 0.065 |  |  |  |
|  | (0.020) | | (0.176) |  |  |  |
| Age |  | |  |  |  |  |
| Age 11 (=1) | 0.063† | | 0.017 | 0.076 | 0.020 | 0.104 |
|  | (0.037) | | (0.088) | (0.114) | (0.102) | (0.119) |
| Age 12 (=1) | 0.028 | | 0.007 | 0.053 | -0.029 | 0.083 |
|  | (0.035) | | (0.080) | (0.106) | (0.092) | (0.110) |
| Age 13 (=1) | -0.002 | | -0.021 | 0.042 | -0.044 | 0.101 |
|  | (0.034) | | (0.080) | (0.102) | (0.091) | (0.110) |
| Age 14 (=1) | -0.038 | | 0.022 | -0.056 | -0.174† | 0.018 |
|  | (0.034) | | (0.076) | (0.099) | (0.089) | (0.108) |
| Age 15 (=1) | Ref | | ref |  |  | - |
|  |  | |  |  |  |  |
| Grade attainment (completed grades) | 0.018* | | 0.006 | 0.023 | 0.032 | -0.010 |
|  | (0.008) | | (0.017) | (0.023) | (0.023) | (0.022) |
| Cognitive test score | 0.001 | | -0.003 | 0.000 | 0.009 | 0.008 |
|  | (0.002) | | (0.006) | (0.007) | (0.007) | (0.008) |
| Mother completed primary school (=1) | 0.012 | | 0.037 | -0.007 | -0.065 | -0.036 |
|  | (0.015) | | (0.037) | (0.046) | (0.046) | (0.045) |
| Father completed primary school (=1) | -0.017 | | -0.017 | 0.017 | -0.011 | -0.003 |
|  | (0.017) | | (0.042) | (0.054) | (0.052) | (0.051) |
| Lives with both parents (=1) | 0.031* | | -0.014 | 0.075† | 0.051 | 0.059 |
|  | (0.014) | | (0.033) | (0.043) | (0.043) | (0.042) |
| Household wealth quintile | -0.005 | | 0.004 | 0.003 | -0.015 | -0.021 |
|  | (0.005) | | (0.011) | (0.015) | (0.015) | (0.014) |
| Constant | 0.688*** | |  |  |  | 0.780*** |
|  | (0.062) | |  |  |  | (0.136) |
|  |  | |  |  |  |  |
| N | 2390 | | 2390 |  |  |  |
| P-value overall F-test | <0.001 | | <0.001 |  |  |  |
| P-value for F-test on treatment arms | 0.001 | | 0.151 |  |  |  |
| P-value for F-test on all interactions with study arms |  | | 0.197 |  |  |  |
| P-value for F-test on interactions with study arm |  | |  | 0.683 | 0.136 | 0.552 |

Notes: Column (1) presents OLS coefficients for the linear probability model of a binary variable (=1) for resurvey at the endline, estimated with robust standard errors. Column (2) is the LPM controlling for the same set of variables (2a) as well as interactions between each right-side variable and a binary 0/1 variable for study arms 2, 3 and 4. *** p<0.001, ** p<0.01, * p<0.05, † p<0.1
