# Supplementary material for: Impacts of multisectoral cash plus programs after four years in an urban informal settlement: Adolescent Girls Initiative-Kenya (AGI-K) randomized trial
Source: PLoS One. 2022 Feb 7;17(2):e0262858. doi: 10.1371/journal.pone.0262858 (PMC8820646; doi:10.1371/journal.pone.0262858)
Supplement: S4 Table — (DOCX) [file pone.0262858.s004.docx]

**S4 Table: Additional estimated intent-to-treat effects on primary outcomes at endline, by study arm**

|  | (1) | | | | | (2) | (3) | (4) | (5) | (6) | (7) | (8) |
| --- | --- | --- | --- | --- | --- | --- | --- | --- | --- | --- | --- | --- |
|  | V-Only Endline  Mean | | | | | VE  Estimate | VEH  Estimate | VEHW  Estimate | VE-VEH-VEHW Pooled  Estimate | VEH vs  VE  (3)-(2) | VEHW vs  VE  (4)-(2) | VEHW vs  VEH  (4)-(3) |
| Ever had sex (=1) | 0.210 | | | | | -0.033 | -0.037 | -0.032 | -0.034† | -0.004 | 0.001 | 0.005 |
| 95% CI |  | | | | | [-0.08, 0.01] | [-0.08, 0.01] | [-0.08, 0.01] | [-0.07, 0.00] | [-0.05, 0.04] | [-0.04, 0.04] | [-0.04, 0.05] |
| P-value |  | | | | | 0.149 | 0.108 | 0.160 | 0.077 | 0.871 | 0.953 | 0.822 |
| Extended controls regression estimate | | |  | | | -0.031 | -0.042† | -0.031 | -0.034† | -0.011 | 0.000 | 0.011 |
| IPW weighted regression estimate | |  | | | | -0.036 | -0.030 | -0.031 | -0.032 | 0.005 | 0.004 | -0.001 |
| Extended controls IPW weighted estimate | | | | |  | -0.034 | -0.036 | -0.031 | -0.033† | -0.002 | 0.003 | 0.005 |
| Ever pregnant (=1) | 0.077 | | | | | -0.014 | 0.006 | -0.018 | -0.009 | 0.021 | -0.004 | -0.025 |
| 95% CI |  | | | | | [-0.04, 0.02] | [-0.03, 0.04] | [-0.05, 0.01] | [-0.03, 0.02] | [-0.01, 0.05] | [-0.03, 0.02] | [-0.05, 0.00] |
| P-value |  | | | | | 0.348 | 0.700 | 0.227 | 0.506 | 0.172 | 0.785 | 0.100 |
| Extended controls regression estimate | | | |  | | -0.014 | 0.002 | -0.019 | -0.010 | 0.016 | -0.005 | -0.021 |
| IPW weighted regression estimate |  | | | | | -0.015 | 0.008 | -0.017 | -0.008 | 0.024 | -0.002 | -0.025 |
| Extended controls IPW weighted estimate | | | | |  | -0.015 | 0.004 | -0.018 | -0.010 | 0.019 | -0.003 | -0.021 |
| Ever given birth (=1) | 0.066 | | | | | -0.023† | 0.007 | -0.023† | -0.013 | 0.030* | 0.000 | -0.030* |
| 95% CI |  | | | | | [-0.05, 0.00] | [-0.02, 0.04] | [-0.05, 0.00] | [-0.04, 0.01] | [0.00, 0.06] | [-0.02, 0.02] | [-0.06, 0.00] |
| P-value |  | | | | | 0.092 | 0.658 | 0.090 | 0.284 | 0.028 | 0.999 | 0.027 |
| Extended controls regression estimate | | | |  | | -0.023† | 0.002 | -0.024† | -0.015 | 0.025† | 0.000 | -0.026† |
| IPW weighted regression estimate |  | | | | | -0.024† | 0.007 | -0.024† | -0.014 | 0.031* | 0.000 | -0.031* |
| Extended controls IPW weighted estimate | | | | |  | -0.024† | 0.002 | -0.025† | -0.016 | 0.026† | -0.001 | -0.026† |
| *Fertility outcomes summary index z-score* | 0.000 | | | | | -0.090 | -0.035 | -0.092† | -0.072 | 0.055 | -0.001 | -0.057 |
| 95% CI |  | | | | | [-0.20, 0.02] | [-0.15, 0.08] | [-0.20, 0.01] | [-0.16, 0.02] | [-0.05, 0.16] | [-0.10, 0.09] | [-0.16, 0.05] |
| P-value |  | | | | | 0.101 | 0.555 | 0.089 | 0.127 | 0.304 | 0.976 | 0.281 |
| Extended controls regression estimate | | |  | | | -0.086 | -0.048 | -0.090† | -0.075 | 0.037 | -0.004 | -0.042 |
| IPW weighted regression estimate |  | | | | | -0.096† | -0.025 | -0.092† | -0.070 | 0.071 | 0.004 | -0.067 |
| Extended controls IPW weighted estimate | | | | |  | -0.093† | -0.041 | -0.092† | -0.075 | 0.052 | 0.001 | -0.051 |

Notes: Sample is N=2,075. The table reports endline means for V-only study arm in column 1 and the estimated ITT effect for each study arm relative to V-only in columns 2–4. Column 5 pools the intervention arms with education into a single treatment indicator. Differences in the estimated ITT effects across study arms are reported in columns 6–8. Column 6 compares the estimates for VEH to VE, column 7 compares VEHW to VE, and column 8 compares VEHW to VEH. For example, the estimate in column 6 for ‘Ever had sex’ (-0.004) is the difference between the estimate for VEH in column 3 (-0.037) and the estimate for VE in column 2 (-0.033). Minor differences in the reported differentials compared to the estimates presented in columns 2–4 are due to rounding. Numbers in square brackets indicate 95% confidence intervals and below them corresponding p-values. Regressions were estimated with robust standard errors and included controls for age and the outcome measured at baseline. The extended control regressions additionally control for baseline measures of grade attainment, cognitive score, mother or father completing primary school, coresidence with both parents, household wealth quintile and whether any missing baseline covariates were imputed using area median. IPW weighted regression estimates reweight results using inverse probability weights described in S3 Text. *** p<0.001, ** p<0.01, * p<0.05, † p<0.1
