# Supplementary material for: Impacts of multisectoral cash plus programs after four years in an urban informal settlement: Adolescent Girls Initiative-Kenya (AGI-K) randomized trial
Source: PLoS One. 2022 Feb 7;17(2):e0262858. doi: 10.1371/journal.pone.0262858 (PMC8820646; doi:10.1371/journal.pone.0262858)
Supplement: S5 Table — (DOCX) [file pone.0262858.s005.docx]

**S5 Table: Additional estimated intent-to-treat effects on secondary outcomes summary measures at endline, by study arm**

|  | | (1) | | | (2) | (3) | (4) | (5) | (6) | (7) |
| --- | --- | --- | --- | --- | --- | --- | --- | --- | --- | --- |
|  | | VE  Estimate | | | VEH  Estimate | VEHW  Estimate | VE-VEH-VEHW Pooled  Estimate | VEH vs  VE  (3)-(2) | VEHW vs  VE  (4)-(2) | VEHW vs  VEH  (4)-(3) |
| *Violence prevention outcomes summary index z-score* | | 0.008 | | | 0.015 | 0.003 | 0.009 | 0.007 | -0.005 | -0.012 |
| 95% CI | | [-0.12, 0.13] | | | [-0.11, 0.14] | [-0.12, 0.13] | [-0.09, 0.11] | [-0.11, 0.13] | [-0.13, 0.12] | [-0.14, 0.11] |
| P-value | | 0.896 | | | 0.812 | 0.966 | 0.868 | 0.913 | 0.932 | 0.849 |
| False discovery rate adjusted q-value | 0.896 | | | | 0.812 | 0.966 | 0.868 | 0.914 | 0.932 | 0.849 |
| Extended controls regression estimate | | -0.013 | | | 0.004 | 0.002 | -0.002 | 0.016 | 0.014 | -0.002 |
| IPW Weighted regression estimate | | -0.007 | | | -0.006 | 0.006 | -0.002 | 0.001 | 0.013 | 0.012 |
| Extended controls IPW weighted estimate | | | | -0.023 | -0.010 | 0.004 | -0.010 | 0.013 | 0.027 | 0.014 |
| *Education outcomes summary index z-score* | | 0.175** | | | 0.096† | 0.108† | 0.126** | -0.080 | -0.067 | 0.013 |
| 95% CI | | [0.07, 0.28] | | | [-0.02, 0.21] | [0.00, 0.22] | [0.03, 0.22] | [-0.18, 0.02] | [-0.16, 0.03] | [-0.09, 0.12] |
| P-value | | 0.002 | | | 0.099 | 0.053 | 0.009 | 0.120 | 0.175 | 0.810 |
| False discovery rate adjusted q-value | | | 0.007 | | 0.132 | 0.072 | 0.012 | 0.255 | 0.350 | 0.849 |
| Extended controls regression estimate | | 0.146** | | | 0.090 | 0.100† | 0.112* | -0.056 | -0.046 | 0.010 |
| IPW Weighted regression estimate | | 0.164** | | | 0.067 | 0.114* | 0.115* | -0.097† | -0.050 | 0.047 |
| Extended controls IPW weighted estimate | | | | 0.140* | 0.067 | 0.105† | 0.104* | -0.073 | -0.035 | 0.038 |
| *Health outcomes summary index z-score* | | 0.095 | | | 0.187** | 0.126* | 0.136** | 0.092 | 0.031 | -0.061 |
| 95% CI | | [-0.03, 0.22] | | | [0.07, 0.31] | [0.00, 0.25] | [0.04, 0.24] | [-0.03, 0.21] | [-0.09, 0.15] | [-0.18, 0.06] |
| P-value | | 0.125 | | | 0.002 | 0.047 | 0.007 | 0.127 | 0.621 | 0.325 |
| False discovery rate adjusted q-value | | | | 0.167 | 0.009 | 0.072 | 0.012 | 0.255 | 0.828 | 0.651 |
| Extended controls regression estimate | | 0.069 | | | 0.167** | 0.119† | 0.119* | 0.098† | 0.050 | -0.048 |
| IPW Weighted regression estimate | | 0.089 | | | 0.167** | 0.132* | 0.130* | 0.078 | 0.044 | -0.035 |
| Extended controls IPW weighted estimate | | | | 0.069 | 0.158** | 0.126* | 0.118* | 0.089 | 0.057 | -0.032 |
| *Wealth creation outcomes summary index z-score* | | 0.158* | | | 0.108† | 0.407*** | 0.225*** | -0.050 | 0.249*** | 0.299*** |
| 95% CI | | [0.04, 0.28] | | | [-0.02, 0.23] | [0.29, 0.53] | [0.12, 0.33] | [-0.17, 0.07] | [0.13, 0.37] | [0.18, 0.42] |
| P-value | | 0.012 | | | 0.088 | 0.000 | 0.000 | 0.428 | 0.000 | 0.000 |
| False discovery rate adjusted q-value | | | | 0.024 | 0.132 | 0.001 | 0.001 | 0.571 | 0.001 | 0.001 |
| Extended controls regression estimate | | 0.128* | | | 0.093 | 0.407*** | 0.210*** | -0.034 | 0.280*** | 0.314*** |
| IPW Weighted regression estimate | | 0.155* | | | 0.096 | 0.400*** | 0.216*** | -0.059 | 0.245*** | 0.304*** |
| Extended controls IPW weighted estimate | | | | 0.129* | 0.088 | 0.398*** | 0.205*** | -0.040 | 0.270*** | 0.310*** |

Notes: Sample is N=2,075. The table reports the estimated ITT effect for the secondary outcome summary measures for each study arm relative to V-only in columns 1–3. Column 4 pools the intervention arms with education into a single treatment indicator. Differences in the estimated ITT effects across study arms are reported in columns 5–7. Column 5 compares the estimates for VEH to VE, column 6 compares VEHW to VE, and column 7 compares VEHW to VEH. Minor differences in the reported differentials compared to the estimates presented in columns 1–3 are due to rounding. Numbers in square brackets indicate 95% confidence intervals and below them corresponding p-values based on robust standard errors. Given we the number of variables tested, to account for multiple hypothesis testing we recalculated statistical significance using the Benjamini and Hochberg (1995) false discovery rates (FDR) and report the adjusted q-values for the main effects compared to the V-only study arm. All regressions included controls for age and the outcome summary measure at baseline. The extended control regressions additionally control for baseline measures of grade attainment, cognitive score, mother or father completing primary school, coresidence with both parents, household wealth quintile and whether any missing baseline covariates were imputed using area median. IPW weighted regression estimates reweight results using inverse probability weights described in S3 Text. To construct the summary indexes, for each individual component outcome (listed in S6 Table) we calculated a z-score based on the mean and standard deviation (SD) of the V-only study arm at endline. Using those, we constructed an inverse covariance weighted index, re-standardizing to be mean 0 and SD 1 (Anderson 2008). *** p<0.001, ** p<0.01, * p<0.05, † p<0.1

References:

Anderson, M. 2008. Multiple Inference and Gender Difference in the Effects of Early Interventions: A Reevaluation of the Abecedarian, Perry Preschool, and Early Training Projects. *Journal of American Statistical Association* 103(484):1481–95.

Benjamini Y, Hochberg Y. 1995. Controlling the false discovery rate: a practical and powerful approach to multiple testing. *Journal of the Royal statistical society: series B* (Methodological); 57:289–300.
