# Supplementary material for: Impacts of multisectoral cash plus programs after four years in an urban informal settlement: Adolescent Girls Initiative-Kenya (AGI-K) randomized trial
Source: PLoS One. 2022 Feb 7;17(2):e0262858. doi: 10.1371/journal.pone.0262858 (PMC8820646; doi:10.1371/journal.pone.0262858)
Supplement: S6 Table — (DOCX) [file pone.0262858.s006.docx]

**S6 Table: Estimated intent-to-treat effects on individual components of secondary outcomes at endline, by study arm**

|  | (1) | | | | | | | | | (2) | (3) | (4) | (5) | (6) | (7) | (8) |
| --- | --- | --- | --- | --- | --- | --- | --- | --- | --- | --- | --- | --- | --- | --- | --- | --- |
|  | V-Only Endline  Mean | | | | | | | | | VE  Estimate | VEH  Estimate | VEHW  Estimate | VE-VEH-VEHW Pooled  Estimate | VEH vs  VE  (3)-(2) | VEHW vs  VE  (4)-(2) | VEHW vs  VEH  (4)-(3) |
| Violence Prevention |  | | | | | | | | |  |  |  |  |  |  |  |
| Experienced violence by a male in the past year (=1) | 0.224 | | | | | | | | | -0.020 | 0.007 | -0.001 | -0.005 | 0.027 | 0.019 | -0.008 |
| 95% CI |  | | | | | | | | | [-0.07, 0.03] | [-0.04, 0.06] | [-0.05, 0.05] | [-0.05, 0.04] | [-0.02, 0.08] | [-0.03, 0.07] | [-0.06, 0.04] |
| P-value |  | | | | | | | | | 0.435 | 0.801 | 0.961 | 0.821 | 0.289 | 0.454 | 0.758 |
| False discovery rate adjusted q-value | | | | |  | | | | | 0.628 | 0.868 | 0.961 | 0.890 | 0.470 | 0.656 | 0.961 |
| Extended controls regression estimate | | | | |  | | | | | -0.020 | 0.004 | -0.001 | -0.005 | 0.025 | 0.020 | -0.005 |
| IPW weighted regression estimate | |  | | | | | | | | -0.017 | 0.011 | -0.002 | -0.002 | 0.028 | 0.015 | -0.013 |
| Extended controls IPW weighted estimate | | | | | | | |  | | -0.017 | 0.009 | -0.001 | -0.003 | 0.026 | 0.016 | -0.010 |
| Gender equitable attitudes z-score^1^ | | 0.000 | | | | | | | | -0.067 | 0.064 | 0.071 | 0.023 | 0.131* | 0.138* | 0.007 |
| 95% CI |  | | | | | | | | | [-0.19, 0.06] | [-0.06, 0.19] | [-0.05, 0.19] | [-0.08, 0.12] | [0.01, 0.25] | [0.02, 0.26] | [-0.11, 0.13] |
| P-value |  | | | | | | | | | 0.292 | 0.310 | 0.261 | 0.661 | 0.035 | 0.027 | 0.909 |
| False discovery rate adjusted q-value | | | | |  | | | | | 0.475 | 0.576 | 0.436 | 0.815 | 0.287 | 0.118 | 0.986 |
| Extended controls regression estimate | | | | | | | | |  | -0.089 | 0.052 | 0.068 | 0.011 | 0.141* | 0.157* | 0.016 |
| IPW weighted regression estimate | |  | | | | | | | | -0.077 | 0.060 | 0.072 | 0.019 | 0.136* | 0.149* | 0.012 |
| Extended controls IPW weighted estimate | | | | | | | | |  | -0.095 | 0.055 | 0.068 | 0.010 | 0.150* | 0.163** | 0.013 |
| Positive gender schooling attitudes z-score | | | | | | | 0.339 | | | 0.019 | -0.015 | -0.046 | -0.014 | -0.034 | -0.065 | -0.031 |
| 95% CI |  | | | | | | | | | [-0.06, 0.10] | [-0.10, 0.07] | [-0.14, 0.04] | [-0.08, 0.06] | [-0.12, 0.05] | [-0.15, 0.02] | [-0.12, 0.06] |
| P-value |  | | | | | | | | | 0.653 | 0.721 | 0.307 | 0.689 | 0.408 | 0.139 | 0.483 |
| False discovery rate adjusted q-value | | | | | | | | |  | 0.772 | 0.853 | 0.444 | 0.815 | 0.488 | 0.451 | 0.961 |
| Extended controls regression estimate | | | | | |  | | | | 0.006 | -0.025 | -0.043 | -0.021 | -0.031 | -0.050 | -0.018 |
| IPW weighted regression estimate | | |  | | | | | | | 0.013 | -0.030 | -0.043 | -0.020 | -0.043 | -0.056 | -0.012 |
| Extended controls IPW weighted estimate | | | | | | | |  | | 0.003 | -0.037 | -0.041 | -0.025 | -0.040 | -0.045 | -0.004 |
| Education |  | | | | | | | | |  |  |  |  |  |  |  |
| Grade attainment [n=2,062] | 9.319 | | | | | | | | | 0.121** | 0.062 | 0.082† | 0.088* | -0.059 | -0.039 | 0.019 |
| 95% CI |  | | | | | | | | | [0.03, 0.21] | [-0.03, 0.16] | [-0.01, 0.17] | [0.01, 0.16] | [-0.14, 0.02] | [-0.12, 0.04] | [-0.06, 0.10] |
| P-value |  | | | | | | | | | 0.008 | 0.191 | 0.067 | 0.025 | 0.164 | 0.312 | 0.637 |
| False discovery rate adjusted q-value | | | | | | | | |  | 0.087 | 0.415 | 0.217 | 0.083 | 0.357 | 0.656 | 0.961 |
| Extended controls regression estimate | | | | |  | | | | | 0.115* | 0.079† | 0.079† | 0.091* | -0.037 | -0.036 | 0.000 |
| IPW weighted regression estimate | |  | | | | | | | | 0.113* | 0.050 | 0.080† | 0.080* | -0.063 | -0.033 | 0.030 |
| Extended controls IPW weighted estimate | | | | | | | |  | | 0.109* | 0.068 | 0.079† | 0.085* | -0.040 | -0.030 | 0.010 |
| Primary school complete (=1) [n=2,063] | | | | | | | | | 0.899 | 0.032† | -0.001 | 0.025 | 0.018 | -0.034† | -0.007 | 0.026 |
| 95% CI |  | | | | | | | | | [0.00, 0.07] | [-0.04, 0.04] | [-0.01, 0.06] | [-0.01, 0.05] | [-0.07, 0.00] | [-0.04, 0.02] | [-0.01, 0.06] |
| P-value |  | | | | | | | | | 0.067 | 0.938 | 0.156 | 0.221 | 0.050 | 0.653 | 0.127 |
| False discovery rate adjusted q-value | | | | | | | | |  | 0.174 | 0.939 | 0.349 | 0.365 | 0.287 | 0.664 | 0.551 |
| Extended controls regression estimate | | | | | | | | |  | 0.017 | -0.009 | 0.024 | 0.010 | -0.026† | 0.007 | 0.033* |
| IPW weighted regression estimate | | | |  | | | | | | 0.028 | -0.010 | 0.025 | 0.014 | -0.038* | -0.003 | 0.035 |
| Extended controls IPW weighted estimate | | | | | | | | |  | 0.016 | -0.012 | 0.023 | 0.009 | -0.028† | 0.007 | 0.035* |
| Enrolled in current school year (=1) [n=2,072] | | | | | | | 0.861 | | | 0.044* | 0.028 | 0.028 | 0.033* | -0.015 | -0.016 | 0.000 |
| 95% CI |  | | | | | | | | | [0.01, 0.08] | [-0.01, 0.07] | [-0.01, 0.07] | [0.00, 0.07] | [-0.05, 0.02] | [-0.05, 0.02] | [-0.04, 0.04] |
| P-value |  | | | | | | | | | 0.023 | 0.156 | 0.161 | 0.047 | 0.370 | 0.377 | 0.997 |
| False discovery rate adjusted q-value | | | | |  | | | | | 0.087 | 0.415 | 0.349 | 0.123 | 0.488 | 0.656 | 0.998 |
| Extended controls regression estimate | | | | |  | | | | | 0.040* | 0.031 | 0.026 | 0.033* | -0.009 | -0.014 | -0.005 |
| IPW weighted regression estimate | | | | |  | | | | | 0.043* | 0.023 | 0.029 | 0.032† | -0.020 | -0.014 | 0.006 |
| Extended controls IPW weighted estimate | | | | | | | | |  | 0.041* | 0.026 | 0.028 | 0.031† | -0.014 | -0.013 | 0.001 |
| Health |  | | | | | | | | |  |  |  |  |  |  |  |
| Knows most fertile period during menstrual cycle (=1) | 0.147 | | | | | | | | | 0.037 | 0.012 | 0.018 | 0.022 | -0.025 | -0.018 | 0.006 |
| 95% CI |  | | | | | | | | | [-0.01, 0.08] | [-0.03, 0.06] | [-0.03, 0.06] | [-0.01, 0.06] | [-0.07, 0.02] | [-0.06, 0.03] | [-0.04, 0.05] |
| P-value |  | | | | | | | | | 0.112 | 0.584 | 0.416 | 0.225 | 0.287 | 0.428 | 0.785 |
| False discovery rate adjusted q-value | | | | |  | | | | | 0.243 | 0.844 | 0.492 | 0.365 | 0.470 | 0.656 | 0.961 |
| Extended controls regression estimate | | | | | |  | | | | 0.033 | 0.010 | 0.018 | 0.020 | -0.023 | -0.015 | 0.007 |
| IPW weighted regression estimate | | | |  | | | | | | 0.039† | 0.008 | 0.018 | 0.022 | -0.031 | -0.021 | 0.010 |
| Extended controls IPW weighted estimate | | | | | | | | |  | 0.036 | 0.007 | 0.017 | 0.020 | -0.028 | -0.019 | 0.010 |
| Knows method of modern contraception^1^ (=1) [n=2,050] | 0.795 | | | | | | | | | 0.055* | 0.073** | 0.068** | 0.065** | 0.017 | 0.012 | -0.005 |
| 95% CI |  | | | | | | | | | [0.01, 0.10] | [0.03, 0.12] | [0.02, 0.11] | [0.03, 0.10] | [-0.02, 0.06] | [-0.03, 0.05] | [-0.05, 0.04] |
| P-value |  | | | | | | | | | 0.021 | 0.002 | 0.004 | 0.001 | 0.413 | 0.563 | 0.812 |
| False discovery rate adjusted q-value | | | | |  | | | | | 0.087 | 0.023 | 0.018 | 0.008 | 0.488 | 0.664 | 0.961 |
| Extended controls regression estimate | | | | |  | | | | | 0.051* | 0.069** | 0.068** | 0.063** | 0.017 | 0.017 | 0.000 |
| IPW weighted regression estimate | |  | | | | | | | | 0.058* | 0.072** | 0.072** | 0.067** | 0.014 | 0.015 | 0.000 |
| Extended controls IPW weighted estimate | | | | | | | |  | | 0.055* | 0.070** | 0.072** | 0.066** | 0.016 | 0.018 | 0.002 |
| SRH myths knowledge z-score^1^ [n=1,880] | | | | | | | 0.000 | | | -0.011 | 0.093 | 0.059 | 0.048 | 0.104 | 0.070 | -0.034 |
| 95% CI |  | | | | | | | | | [-0.14, 0.12] | [-0.04, 0.22] | [-0.07, 0.19] | [-0.06, 0.16] | [-0.02, 0.23] | [-0.06, 0.20] | [-0.16, 0.09] |
| P-value |  | | | | | | | | | 0.865 | 0.160 | 0.384 | 0.388 | 0.102 | 0.284 | 0.603 |
| False discovery rate adjusted q-value | | | | |  | | | | | 0.917 | 0.415 | 0.492 | 0.560 | 0.287 | 0.656 | 0.961 |
| Extended controls regression estimate | | | | |  | | | | | -0.029 | 0.069 | 0.056 | 0.033 | 0.098 | 0.085 | -0.013 |
| IPW weighted regression estimate | | | |  | | | | | | -0.011 | 0.083 | 0.058 | 0.044 | 0.094 | 0.070 | -0.024 |
| Extended controls IPW weighted estimate | | | | | | | |  | | -0.027 | 0.065 | 0.056 | 0.032 | 0.092 | 0.083 | -0.009 |
| General self-efficacy z-score | 0.630 | | | | | | | | | -0.005 | 0.028 | -0.025 | -0.001 | 0.033 | -0.020 | -0.053 |
| 95% CI |  | | | | | | | | | [-0.09, 0.08] | [-0.06, 0.12] | [-0.12, 0.07] | [-0.07, 0.07] | [-0.05, 0.12] | [-0.11, 0.07] | [-0.14, 0.04] |
| P-value |  | | | | | | | | | 0.916 | 0.535 | 0.599 | 0.985 | 0.458 | 0.664 | 0.255 |
| False discovery rate adjusted q-value | | | | |  | | | | | 0.917 | 0.844 | 0.650 | 0.986 | 0.497 | 0.664 | 0.829 |
| Extended controls regression estimate | | | | |  | | | | | -0.016 | 0.025 | -0.027 | -0.006 | 0.041 | -0.010 | -0.052 |
| IPW weighted regression estimate | | | |  | | | | | | -0.015 | 0.009 | -0.024 | -0.010 | 0.024 | -0.009 | -0.033 |
| Extended controls IPW weighted estimate | | | | | | | | |  | -0.023 | 0.012 | -0.024 | -0.012 | 0.034 | -0.002 | -0.036 |
| Condom use self-efficacy z-score^1^ [n=1,875] | | | | | | | | 0.000 | | 0.032 | 0.135* | 0.073 | 0.080 | 0.103 | 0.041 | -0.062 |
| 95% CI |  | | | | | | | | | [0.10, 0.16] | [0.01, 0.26] | [-0.06, 0.20] | [-0.03, 0.19] | [-0.02, 0.23] | [-0.09, 0.17] | [-0.19, 0.06] |
| P-value |  | | | | | | | | | 0.624 | 0.040 | 0.268 | 0.138 | 0.110 | 0.529 | 0.336 |
| False discovery rate adjusted q-value | | | | | | | | |  | 0.772 | 0.173 | 0.436 | 0.300 | 0.287 | 0.664 | 0.875 |
| Extended controls regression estimate | | | | |  | | | | | 0.017 | 0.119† | 0.068 | 0.068 | 0.102 | 0.051 | -0.051 |
| IPW weighted regression estimate | | |  | | | | | | | 0.020 | 0.132* | 0.082 | 0.079 | 0.112† | 0.062 | -0.050 |
| Extended controls IPW weighted estimate | | | | | | | |  | | 0.007 | 0.122† | 0.077 | 0.069 | 0.115† | 0.071 | -0.045 |
| Wealth creation |  | | | | | | | | |  |  |  |  |  |  |  |
| Financial literacy z-score | 0.379 | | | | | | | | | 0.122* | 0.024 | 0.301*** | 0.149** | -0.098† | 0.180** | 0.278*** |
| 95% CI |  | | | | | | | | | [0.01, 0.23] | [-0.09, 0.13] | [0.20, 0.41] | [0.06, 0.24] | [-0.20, 0.01] | [0.08, 0.28] | [0.17, 0.38] |
| P-value |  | | | | | | | | | 0.026 | 0.671 | 0.000 | 0.001 | 0.069 | 0.001 | 0.000 |
| False discovery rate adjusted q-value | | | | |  | | | | | 0.087 | 0.853 | 0.001 | 0.008 | 0.287 | 0.008 | 0.001 |
| Extended controls regression estimate | | | | |  | | | | | 0.102† | 0.010 | 0.300*** | 0.138** | -0.092† | 0.198*** | 0.290*** |
| IPW weighted regression estimate | | |  | | | | | | | 0.120* | 0.017 | 0.291*** | 0.142** | -0.103† | 0.171** | 0.274*** |
| Extended controls IPW weighted estimate | | | | | | | | |  | 0.104† | 0.009 | 0.289*** | 0.133** | -0.095† | 0.186*** | 0.280*** |
| Saved money in the past six months (=1) | 0.423 | | | | | | | | | 0.046 | 0.064* | 0.129*** | 0.080** | 0.018 | 0.083** | 0.065* |
| 95% CI |  | | | | | | | | | [-0.02, 0.11] | [0.00, 0.12] | [0.07, 0.19] | [0.03, 0.13] | [-0.04, 0.08] | [0.02, 0.14] | [0.01, 0.12] |
| P-value |  | | | | | | | | | 0.142 | 0.039 | 0.000 | 0.002 | 0.550 | 0.006 | 0.032 |
| False discovery rate adjusted q-value | | | | |  | | | | | 0.264 | 0.173 | 0.001 | 0.008 | 0.551 | 0.042 | 0.210 |
| Extended controls regression estimate | | | | |  | | | | | 0.035 | 0.061* | 0.128*** | 0.075** | 0.026 | 0.094** | 0.067* |
| IPW weighted regression estimate | | | | | | |  | | | 0.044 | 0.058† | 0.129*** | 0.077** | 0.014 | 0.086** | 0.071* |
| Extended controls IPW weighted estimate | | | | | | | |  | | 0.035 | 0.058† | 0.128*** | 0.074** | 0.023 | 0.093** | 0.070* |

Notes: Sample is N=2,075 unless otherwise indicated. The table reports endline means for V-only study arm in column 1 and the estimated ITT effect for the secondary outcome component measures for each study arm relative to V-only in columns 2–3. Column 5 pools the intervention arms with education into a single treatment indicator. Differences in the estimated ITT effects across study arms are reported in columns 6–8. Column 6 compares the estimates for VEH to VE, column 7 compares VEHW to VE, and column 8 compares VEHW to VEH. Minor differences in the reported differentials compared to the estimates presented in columns 2–4 are due to rounding. Numbers in square brackets indicate 95% confidence intervals and below them corresponding p-values based on robust standard errors. Given we the number of variables tested, to account for multiple hypothesis testing we recalculated statistical significance using the Benjamini and Hochberg (1995) false discovery rates (FDR) and report the adjusted q-values for the main effects compared to the V-only study arm. All regressions included controls for age and the outcome summary measure at baseline. The extended control regressions additionally control for baseline measures of grade attainment, cognitive score, mother or father completing primary school, coresidence with both parents, household wealth quintile and whether any missing baseline covariates were imputed using area median. IPW weighted regression estimates reweight results using inverse probability weights described in S3 Text. *** p<0.001, ** p<0.01, * p<0.05, † p<0.1

^1^ No baseline control for outcome variable available. *** p<0.001, ** p<0.01, * p<0.05, † p<0.1

References:

Benjamini Y, Hochberg Y. 1995. Controlling the false discovery rate: a practical and powerful approach to multiple testing. *Journal of the Royal statistical society: series B* (Methodological); 57:289–300.
