# Supplementary material for: Impacts of multisectoral cash plus programs after four years in an urban informal settlement: Adolescent Girls Initiative-Kenya (AGI-K) randomized trial
Source: PLoS One. 2022 Feb 7;17(2):e0262858. doi: 10.1371/journal.pone.0262858 (PMC8820646; doi:10.1371/journal.pone.0262858)
Supplement: S7 Table — (DOCX) [file pone.0262858.s007.docx]

**S7 Table: Endline outcome variable definitions**

| Variable | Survey items | Survey instrument |
| --- | --- | --- |
| **Primary outcomes** |  |  |
| *Ever had sex*  =1 if reported an age or don’t know; 0 if reported has never had sexual intercourse. | How old were you when you had sexual intercourse for the very first time? | Individual |
| *Ever pregnant*  =1 if answered yes to one or more of these questions; 0 otherwise. | Have you ever given birth?  Have you ever given birth to a boy or girl who was born alive but later died?  Are you pregnant now?  Have you ever been pregnant when you did not want to be?  Have you ever had a pregnancy that miscarried, or ended in a stillbirth? | Individual |
| *Ever given birth*  =1 if reported yes to any of these questions; 0 otherwise. | Have you ever given birth?  Have you ever given birth to a boy or girl who was born alive but later died? | Individual |
| *Fertility outcomes summary index z-score* | Constructed using *ever had sex*, *ever pregnant,* and *ever given birth* |  |
| *HSV-2 prevalence*  =1 if tested positive; 0 if tested negative or indeterminate. | Tested positive for HSV-2 (prevalence) | Individual biomarker |
| *HSV-2 incidence*  =1 if tested negative in 2017 and positive in 2019; 0 if tested negative in 2017 and 2019. | Among girls who tested negative for HSV-2 in 2017, tested positive for HSV-2 (incidence between 2017–2019) | Individual biomarker |
| **Violence prevention and gender attitudes domain** | |  |
| *Experienced violence by a male in the past year*  =1 if answered yes to any one of the 15 examples; 0 otherwise. | Has any male done any of the following things to you in the past year:   1. Say or do something to humiliate you in front of others 2. Threaten to hurt or harm you or someone close to you 3. Insult you or make you feel bad about yourself 4. Push you, shake you, or throw something at you 5. Slap you 6. Twist your arm or pull your hair 7. Punch you with his fist or something that could hurt you 8. Kick you, drag you, or beat you up 9. Try to choke you or burn you on purpose 10. Threatened to attack you with a knife or other weapon 11. Attacked you with a weapon 12. Touched you in a sexual way (e.g., kissing, grabbing, or fondling), when you did not want them to 13. Try to have sexual intercourse with you when you did not want to but did not succeed 14. Physically forced you to have sexual intercourse even when you did not want to 15. Forced you to perform sexual acts when you did not want to | Individual |
| *Gender equitable attitudes score*  Summative score ranging from 0–10 with one point for each answer corresponding to the more gender equitable response as shown. | Agree or disagree with the statements:   1. Girls should avoid playing sports with boys because they get hurt easily (disagree=1) 2. Boys should be raised tough so they can overcome any difficulty in life (disagree=1) 3. Girls should avoid raising their voice to be lady like (disagree=1) 4. Boys should always defend themselves even if it means fighting (disagree=1) 5. Girls are expected to be humble (disagree=1) 6. Girls should always fight back if boys try to take advantage of them (agree=1) 7. Girls need their parents’ protection more than boys (disagree=1) 8. Boys should be able to show their feelings without fear of being teased (agree=1) 9. Boys who behave like girls are considered weak (disagree=1) 10. It's important for boys to show they are tough (disagree=1) | Individual |
| *Gender equitable schooling attitudes*  Summative score ranging from 0–4 with one point for each answer corresponding to the more gender equitable response as shown. | Agree or disagree with the statements:   1. It is as important for girls to complete secondary school as it is for boys (agree=1) 2. When a family cannot afford to send all children to school, it is better to send boys than girls (disagree=1) 3. A 16-year-old girl should get married when she finds an appropriate partner, even if she is still in school (disagree=1) 4. Girls are as intelligent as boys (agree=1) | Individual |
| *Violence prevention outcomes summary index z-score* | Constructed using *experienced violence by a male in the past year*, *gender equitable attitudes score*, and *gender equitable schooling attitudes* |  |
|  |  |  |
| **Education domain** |  |  |
|  |  |  |
| *Grade attainment*  Number of completed grades, 0–12. | What was the highest level of school you attended: primary or secondary? What is the highest class you completed at primary school? What is the highest class you completed at secondary school? | Individual |
| *Primary school complete*  =1 if completed; 0 otherwise. | Has completed primary school (class 8) | Individual |
| *School enrollment*  =1 if enrolled; 0 otherwise. | Have you attended school at any time during [the 2017 school year]? | Individual |
| *Conditional primary school completion*  =1 if completed grade 8; 0 otherwise. Variable only defined for those who had completed grade 6 or 7 at baseline. | Respondents who had completed class 6 or 7 (but not yet class 8) at baseline had completed class 8 at follow-up | Individual |
| *Transition to secondary school*  =1 if enrolled in secondary; 0 otherwise. Variable only defined for those who had completed grade 6, 7 or 8 at baseline but had not yet enrolled in secondary. | Respondents who had completed class 6, 7 or 8 (but had not enrolled in secondary) at baseline had enrolled in secondary at follow-up | Individual |
|  |  |  |
| *Literate in Swahili and English*  =1 if read all four sentences correctly without interruption; 0 otherwise.  (Not an outcome variable but summarized in baseline tables.) | Read aloud the following sentences in Swahili:   1. Ukulima ni kazi ngumu. 2. Mtoto anasoma kitabu.   Sentences in English:   1. Parents love their children. 2. Farming is hard work. | Individual literacy assessment |
| *Education outcomes summary index z-score* | Constructed using *grade attainment*, *primary school complete*, and *school enrollment* |  |
|  |  |  |
| **Health domain** |  |  |
| *Knows most fertile period during menstrual cycle*  =1 if knows; 0 otherwise. | From one menstrual period to the next, are there certain days when a woman is more likely to become pregnant if she has sexual relations? Is this time just before her period begins, during her period, right after her period has ended, or two weeks after her period? (two weeks after her period=1) | Individual |
| *Knows method of modern contraception*  =1 if spontaneously named a modern contraceptive method; 0 otherwise. | I would like to talk about family planning – the various ways or methods that a couple can use to delay or avoid a pregnancy. Which ways or methods have you heard about? Pill, IUD, injectables, implants, male condom, female condom, emergency contraception | Individual |
| *SRH knowledge score*  Summative score ranging from 0–7 with one point for each correct statement about SRH correctly identified as a myth or a fact. | State if the following are facts or myths:   1. One cannot get pregnant with one sexual act (myth=1) 2. Contraceptives are for married women (myth=1) 3. Use two condoms for double protection (myth=1) 4. Menstrual blood means a woman is dirty (myth=1) 5. Contraceptive use is harmful for health (myth=1) 6. Contraceptive pills make women barren (myth=1) 7. Sexual feelings are normal (fact=1) | Individual |
| *General self-efficacy score*  Summative score ranging from 0–6 with one point for each statement agreed to. | I am going to read you some statements, please tell me if you agree or disagree with them:   1. I can always manage to solve difficult problems if I try hard enough. (agree=1) 2. I am confident that I could handle unexpected events very well. (agree=1) 3. Because of the help I can get, I know how to manage unexpected situations. (agree=1) 4. I can solve most problems if I make the necessary effort. (agree=1) 5. If I am in trouble, I can usually think of a solution. (agree=1) 6. I can usually handle any situation that comes my way. (agree=1) | Individual  Alpha: 0.59 |
| *Condom use self-efficacy score*  Summative score ranging from 5–25 based on responses to the questions scored 1–5 each. | I am going to read you some statements, please tell me if you are very unsure=1, somewhat unsure=2, somewhat sure=3, sure=4, very sure=5:   1. I could carry a condom with me in case I needed one 2. I could use a condom each time I and my partner had sex 3. I could talk about using a condom if I were unsure about my partner's feelings on condoms 4. I could talk a partner into using a condom when we have sexual intercourse 5. I could say no to sex if my partner refused to use a condom | Individual  Alpha: 0.85 |
| *Health outcomes summary index z-score* | Constructed using *knows most fertile period during menstrual cycle*, *knows method of modern contraception*, *SRH knowledge score*, *general self-efficacy score*, and *condom use self-efficacy score* |  |
|  |  |  |
| **Wealth creation domain** |  |  |
|  |  |  |
| *Financial literacy score*  Summative score ranging from 0–10 with one point for each correct or savings-oriented answer. | I’m going to read you a story and then ask you some questions about the story: "Each week, Anna sits down and plans what she will earn and spend in the next week. She writes down all the places where she will get money and all the things she will spend it on. Then she is able to see if she has enough money for all of what she wants to buy."   1. Do you have such a plan? 2. What would you call that kind of plan?   I’m going to read you another story and then ask you some questions about it: ''Imani is 17 and and lives with her mother and her younger sister. Her older sister Mary is married and lives in another town, three hours away. Mary just had a baby boy, and Imani is eager to visit her sister. Imani will need to save money for transport and a small gift for the baby. A cute little hat would be perfect! But she can’t take money from her savings because she is saving that money to start her own business. Imani’s dream is to start a small catering business. Hopefully, her neighbor will employ her to work extra days in her hotel so she can get the money she needs for her trip."   1. What is one of Imani's short term financial goals? 2. What is Imani’s long term financial goal? 3. What is one formal way of saving your money? 4. What is one informal way of saving your money? 5. Grace would like to buy a new notebook for the next school term which starts in eight weeks. If the notebook costs KSH 100 and she can save KSH10 each week, will she reach her goal? 6. In the situation I described in the previous question, if Grace figured out how much she needed to save each week, and for how many weeks she needed to save in order to reach her goal, what would that be called? 7. If Grace discovered that she couldn’t reach her goal with that plan, what changes could she make so she would still reach her goal? 8. Do you agree or disagree with the following statement: “Only people with a lot of money can save”? | Individual |
|  |  |  |
| *Saved money in the past six months*  =1 if yes; 0 otherwise. | In the past six months, have you saved, or put money aside to use at a later time? | Individual |
| *Wealth creation outcomes summary index z-score* | Constructed using *financial literacy score* and *saved money in the past six months* |  |
|  |  |  |
| **Household-level domain** |  |  |
| *Household expects girl to complete secondary school*  =1 if highest level is secondary school or higher, 0 if not | What is the highest level of school that you expect [NAME] to complete? | Household |
| *Household wealth quintile*  Quintile of the first component of the PCA with 1 representing the lowest and 5 the highest. | Principal components analysis (PCA) estimated using the following ten items:     1. Does this household own any livestock, herds, or other farm animals or poultry? 2. Does any member of this household own any agricultural land? 3. Does your household have a television? 4. Does your household have a clock or watch? 5. Does your household have a mosquito net? 6. How many rooms in this household are used for sleeping? 7. In the last one month, was there a day that the household went without food because there wasn't enough food in the household? 8. Does your household have enough savings or something(s) to sell if you need 1000 shillings? 9. Does your household have enough savings or something(s) to sell if you need 5000 shillings? 10. Does your household have enough savings or something(s) to sell if you need 10000 shillings? | Household |
|  |  |  |
