# Supplementary material for: Impacts of multisectoral cash plus programs after four years in an urban informal settlement: Adolescent Girls Initiative-Kenya (AGI-K) randomized trial
Source: PLoS One. 2022 Feb 7;17(2):e0262858. doi: 10.1371/journal.pone.0262858 (PMC8820646; doi:10.1371/journal.pone.0262858)
Supplement: S1 Text — (DOCX) [file pone.0262858.s008.docx]

**S1 Text: Note on attrition weight construction (Inverse Probability Weights-IPW)**

We calculated inverse probability weights (IPW) for individual- and household-level survey variables separately as follows. First, we constructed approximately 60 variables from each baseline survey (imputing the less than 1% of missing values with local area medians). In addition to variables in S2 Table, these included other individual level indicators (such as measures of residential mobility) and a wide range of housing characteristics and ownership of household-level assets reported in Austrian et al. (2015). Second, we ran bivariate regressions on an indicator of being interviewed at the four-year follow-up for each variable (separately for the V-only sample and for the VE, VEH and VEHW sample). We retained for potential inclusion in the IPW construction all variables significant at 10%. Third, each variable, along with indicators for age in years was interacted with a binary indicator of treatment in VE, VEH and VEHW (as in S3 Table) so that the weights could be calculated separately for each study arm. Fourth, we estimated the probability of being interviewed on this set of baseline predictors. To account for collinearity between predictors, the baseline predictor set was further limited by conducting stepwise selection of variables with backward elimination and using the adjusted R^2^ as the information criteria. Indicators for study arm and age were fixed in the regressions. At each step, the iterative procedure removes from the model the predictor that most improves the information criterion until there is no variable whose removal improves it. We implemented this using ‘vselect’ in Stata (Lindsey and Sheather 2010). Last, using the final model for each site and survey type, we predicted for each observation the probability of having been re-interviewed and constructed the IPW. Sample weights in Kibera for the individual analyses ranged from 0.81 to 2.28 (25^th^ percentile 1.04, 50^th^ 1.12, 75^th^ 1.23).

References:

Austrian, K, Muthengi, E, Riley, T, Mumah, J, Kabiru C and Abuya B. 2015. AGI-K Baseline Report. Nairobi: Population Council.

Lindsey, C. and S. Sheather. 2010. “Variable selection in linear regression.” *The Stata Journal*, 10(4): 650–669.

**S3 Table: Baseline correlates of endline survey response, by study arm**

| Dependent variable: Interviewed at endline (=1) | | (1) | (2a) | (2b) | (2c) | (2d) |
| --- | --- | --- | --- | --- | --- | --- |
|  |  | | X | Study arm 2*X | Study arm 3*X | Study arm 4*X |
| Study arms: V-only (ref) |  | |  |  |  |  |
| VE (=1) | 0.083*** | | -0.139 |  |  |  |
|  | (0.021) | | (0.187) |  |  |  |
| VEH (=1) | 0.068*** | | -0.075 |  |  |  |
|  | (0.021) | | (0.170) |  |  |  |
| VEHW (=1) | 0.091*** | | 0.065 |  |  |  |
|  | (0.020) | | (0.176) |  |  |  |
| Age |  | |  |  |  |  |
| Age 11 (=1) | 0.063† | | 0.017 | 0.076 | 0.020 | 0.104 |
|  | (0.037) | | (0.088) | (0.114) | (0.102) | (0.119) |
| Age 12 (=1) | 0.028 | | 0.007 | 0.053 | -0.029 | 0.083 |
|  | (0.035) | | (0.080) | (0.106) | (0.092) | (0.110) |
| Age 13 (=1) | -0.002 | | -0.021 | 0.042 | -0.044 | 0.101 |
|  | (0.034) | | (0.080) | (0.102) | (0.091) | (0.110) |
| Age 14 (=1) | -0.038 | | 0.022 | -0.056 | -0.174† | 0.018 |
|  | (0.034) | | (0.076) | (0.099) | (0.089) | (0.108) |
| Age 15 (=1) | Ref | | ref |  |  | - |
|  |  | |  |  |  |  |
| Grade attainment (completed grades) | 0.018* | | 0.006 | 0.023 | 0.032 | -0.010 |
|  | (0.008) | | (0.017) | (0.023) | (0.023) | (0.022) |
| Cognitive test score | 0.001 | | -0.003 | 0.000 | 0.009 | 0.008 |
|  | (0.002) | | (0.006) | (0.007) | (0.007) | (0.008) |
| Mother completed primary school (=1) | 0.012 | | 0.037 | -0.007 | -0.065 | -0.036 |
|  | (0.015) | | (0.037) | (0.046) | (0.046) | (0.045) |
| Father completed primary school (=1) | -0.017 | | -0.017 | 0.017 | -0.011 | -0.003 |
|  | (0.017) | | (0.042) | (0.054) | (0.052) | (0.051) |
| Lives with both parents (=1) | 0.031* | | -0.014 | 0.075† | 0.051 | 0.059 |
|  | (0.014) | | (0.033) | (0.043) | (0.043) | (0.042) |
| Household wealth quintile | -0.005 | | 0.004 | 0.003 | -0.015 | -0.021 |
|  | (0.005) | | (0.011) | (0.015) | (0.015) | (0.014) |
| Constant | 0.688*** | |  |  |  | 0.780*** |
|  | (0.062) | |  |  |  | (0.136) |
|  |  | |  |  |  |  |
| N | 2390 | | 2390 |  |  |  |
| P-value overall F-test | <0.001 | | <0.001 |  |  |  |
| P-value for F-test on treatment arms | 0.001 | | 0.151 |  |  |  |
| P-value for F-test on all interactions with study arms |  | | 0.197 |  |  |  |
| P-value for F-test on interactions with study arm |  | |  | 0.683 | 0.136 | 0.552 |

Notes: Column (1) presents OLS coefficients for the linear probability model of a binary variable (=1) for resurvey at the endline, estimated with robust standard errors. Column (2) is the LPM controlling for the same set of variables (2a) as well as interactions between each right-side variable and a binary 0/1 variable for study arms 2, 3 and 4. *** p<0.001, ** p<0.01, * p<0.05, † p<0.1
