# Supplementary material for: Impacts of multisectoral cash plus programs after four years in an urban informal settlement: Adolescent Girls Initiative-Kenya (AGI-K) randomized trial
Source: PLoS One. 2022 Feb 7;17(2):e0262858. doi: 10.1371/journal.pone.0262858 (PMC8820646; doi:10.1371/journal.pone.0262858)
Supplement: S2 Text — (DOCX) [file pone.0262858.s009.docx]

**S2 Text: HSV-2 Testing Procedures**

HSV-2 biological specimens were collected by trained HIV Testing Services counselors from girls 15 years old and older in 2017 and 2019. For each girl a 2mL sample of whole blood was collected via finger prick and stored in a vacutainer. This protocol for field-testing for HSV-2 has been used in various settings in sub-Saharan Africa (van Dyck et al. 2004; Sudfeld et al. 2013; Hewett et al. 2017). Samples were brought daily by the survey team leaders to the study office where at the end of each day the partner laboratory collected them, transported them to the laboratory and derived serum from the whole blood. The serum was tested using the Kalon ELISA antibody test for which the sensitivity and specificity have been found to be high in clinical evaluations when compared to the Western Blot test in similar settings (Laeyendecker et al. 2004). Laboratory testing procedures were validated prior to testing specimens in both the 2017 and 2019 survey rounds to assure the quality of protocols. Indeterminate results were not retested. After specimen collection, all participants were provided information about HSV-2 detection, symptoms, safe sex practices and treatment options. Respondents were provided vouchers with identification numbers to receive their test results and further counselling by the AGI-K study’s biomarker coordinator at the study office. Girls were notified by text message when their test results were available. The results were available at the AGI-K study office for one month after the completion of data collection. Girls with indeterminate results were referred to one of the study referral health facilities to be retested free of charge.

References:

Hewett, P. C., K. Austrian, E. Soler-Hampejsek, J. R. Behrman, F. Bozzani and N. A. Jackson-Hachonda. 2017. Cluster randomized evaluation of Adolescent Girls Empowerment Programme (AGEP): study protocol. *BMC Public Health* **17**(1): 386.

Laeyendecker, O., C. Henson, R. H. Gray, R. H.-N. Nguyen, B. J. Horne, M. J. Wawer, D. Serwadda, N. Kiwanuka, R. A. Morrow and W. Hogrefe. 2004. Performance of a commercial, type-specific enzyme-linked immunosorbent assay for detection of herpes simplex virus type 2-specific antibodies in Ugandans. *Journal of clinical microbiology* **42**(4): 1794–6.

Sudfeld, C. R., P. C. Hewett, N. N. Abuelezam, S. Chalasani, E. Soler-Hampejsek, C. A. Kelly and B. S. Mensch. 2013. Herpes simplex virus type 2 cross-sectional seroprevalence and the estimated rate of neonatal infections among a cohort of rural Malawian female adolescents. *Sexually Transmitted Infections* **89**(7): 561–7.

van Dyck, E., A. Buve, H. A. Weiss, J. R. Glynn, D. W. Brown, B. De Deken, J. Parry and R. J. Hayes. 2004. Performance of commercially available enzyme immunoassays for detection of antibodies against herpes simplex virus type 2 in African populations. *J Clin Microbiol* **42**(7): 2961–5.
