# Supplementary material for: Impacts of multisectoral cash plus programs after four years in an urban informal settlement: Adolescent Girls Initiative-Kenya (AGI-K) randomized trial
Source: PLoS One. 2022 Feb 7;17(2):e0262858. doi: 10.1371/journal.pone.0262858 (PMC8820646; doi:10.1371/journal.pone.0262858)
Supplement: S1 File — (DOCX) [file pone.0262858.s010.docx]

1. **Title Page**

Project title: Adolescent Girls Initiative - Kenya

Principal Investigators: Karen Austrian, PhD^1^: [kaustrian@popcouncil.org](mailto:kaustrian@popcouncil.org)

Co-Investigators: Erica Soler-Hampejsek, PhD^2^

Beth Kangwana, PhD^1^

Benta Abuya, PhD^3^

Joyce Mumah, PhD^3^

John Maluccio, PhD^4^

| ^1^ Population Council – Kenya  Avenue 5, 3^rd^ Floor  Rose Avenue  Nairobi, Kenya  +254-20-271-3480 | ^2^ Independent Consultant |
| --- | --- |
| ^3^ African Population and Health Research Center APHRC Campus, 2nd Floor  Manga Close, off Kirawa Road  P.O. Box 10787-00100 GPO  Nairobi, Kenya  + 254 (20) 400 1000 | ^4^ Middlebury College  Department of Economics  Middlebury, VT 05753  +1 (802) 443-5941 |
|  |  |

Project location: Wajir County & Nairobi County, Kenya

Proposed project dates: October 1^st^, 2014 to October 31^st^, 2019

Submitted to the IRB on August 14^th^, 2014

Karen Austrian Date August 21, 2014

**Study Protocol Approvals:**

**Population Council Institutional Review Board**

Original submission: August 14, 2014

Protocol approval: September 25, 2014

Amendment #1 approval: May 2, 2016

Amendment #2 approval: March 10, 2017

Amendment #3 approval: April 10^th^, 2018

1. **Summary of proposed research**

Overall Goal of the Project

The Adolescent Girls Initiative – Kenya (AGI-K), will deliver multi-sectoral interventions for 6,200 girls ages 11-14 in two marginalized areas of Kenya: 1) Nairobi slums and 2) Wajir County in Northeastern Kenya. The program will be implemented by a consortium of partners led by Population Council. The research partners include the African Population and Health Research Center (APHRC) and Itad. Save the Children will be the implementing partner in Wajir, while Plan International will oversee the intervention implementation in Nairobi. The interventions, which will run for two years, will comprise a combination of girl-level, household-level and community-level interventions across four sectors: education, health, violence prevention and wealth creation.

The overall goal of the interventions is to reduce adolescent fertility in the long-run by improving the wellbeing of girls in programme sites. A randomized controlled trial will be used to compare the impact of four different packages of interventions. In the Nairobi study, randomization to the four study arms will occur at the individual level. In Nairobi the design also includes an external control site. In Wajir, approximately 80 clusters (school-catchment areas) will be randomized to the four study arms. The research design will use both qualitative and quantitative methods to evaluate this impact. The quantitative study will consist of a baseline survey before the intervention is implemented, randomization to intervention packages, a Midterm survey with the same girls after the two-year intervention, and an endline survey two years post-intervention. Biomarker data, including testing for anaemia, Human Immunodeficiency Virus (HIV) and Herpes Simplex Virus 2 (HSV-2), will be collected at midterm and endline in the Nairobi sample. In-depths interviews will be conducted with a sample of program participants and key stakeholders halfway through the intervention and one-year post-intervention to better understand the causal mechanisms and the strengths and weaknesses of the intervention implementation. In addition, careful costing data will be collected to understand the cost of each intervention.

Risks and Benefits

Primary ethical concerns of this study include a) that participation in the study may put the respondent at risk of psychological trauma related to recounting violent or traumatic experiences, b) that this study includes minors, below the age of 18, and c) a risk of breach of confidentiality due to the collection of contact information and identifying information for future survey rounds. These risks are perceived to be minimal and steps will be taken to reduce the risks to participants.

Participants will receive no compensation for their participation in the study. In intervention sites, participants will benefit directly or indirectly from project interventions. All participants will be providing information that will directly inform the development of the interventions for adolescent girls in Kenya.

1. **Study Relationships/Related Project**

This project included a 9-month inception phase, from January to September, 2014. A protocol exemption for the inception phase was obtained on February 19th, 2014. This new protocol covers the entire implementation phase of the project, from October 1st, 2014 to October 31st, 2019. There are four sub-awardee institutions for this project. Additional information regarding each institution is provided below.

The African Population and Health Research Center, Inc. (APHRC) is an international non-profit, research institute that conducts high-quality research on urbanization, population, health and education issues facing sub-Saharan Africa. The Center was established in 1995 as a program of the Population Council and became an autonomous research institution in 2001. The role of APHRC in the consortium is to: 1) Collaborate on research design with Population Council, 2) Make recommendations on how the interventions will need to be designed and implemented given the research design, 3) Assist in development of survey tools and other research instruments and 4) Contribute to analysis and publications. The key personnel for AGI-K will be Caroline Kabiru, Benta Abuya and Joyce Mumah.

Itad is a UK-based independent consultancy established in 1984. They have particular strengths in: evaluating large, multi-disciplinary programmes, policies and organisations; designing and implementing complex monitoring and evaluation (M&E) systems; and training, capacity building and lesson learning in M&E. The role of Itad in the consortium is to: 1) Conduct the cost benefit analysis and cost effectiveness component of study, including input into the research design and tools to ensure that appropriate costing data is collected, and 2) Provide expertise in value for money through the project period. The key technical personnel for Itad on AGI-K will be Valsa Shah.

Save the Children is the world’s leading independent organization for children, creating lasting change in the lives of children in need in 120 countries around the world. They have been operational in Kenya since the 1950s, providing support to children through developmental and humanitarian relief programmes delivered both directly and through local partners. The role of Save the Children in the AGI-K consortium is to: 1) Lead implementation (either directly or through management of local partners) of the interventions in Wajir County, and 2) Supervise and monitor interventions in Wajir County, to ensure that the interventions are implemented with strict adherence to the research design. The key personnel for Save the Children on AGI-K will be Joyce Kimani.

Plan International (Plan) is an International Humanitarian, child-centred development organisation without religious, political or governmental affiliation. Plan has worked in Kenya since 1982, helping poor children to access their rights to health, education, sanitation and protection.  Plan strives to reduce poverty, raise children’s voices, support orphans and vulnerable children, and attend to those with special needs, such as the disabled. Plan’s role in the AGI-K consortium is to: 1) Lead implementation of the interventions in Nairobi County, and 2) Supervise and monitor interventions in Nairobi County to ensure that the interventions are implemented with strict adherence to the research design. The key personnel for Plan on AGI-K will be Anthony Ombara.

1. **Background**

Many adolescent girls in Kenya face considerable risks and vulnerabilities that affect their education status, health and general wellbeing. Therefore, it is critical to intervene at a time when girls are experiencing a myriad of challenges, but before those challenges have resulted in outcomes that may be irreversible. There is a wide range of research that has shown the benefits of education for girls. Documented benefits include delaying marriage, lowering family size, positive effects on a woman’s children and economic benefits to a woman, her family, and community. However, in addition, new thinking on education also posits that education alone is not enough to achieve ‘empowerment’, and that girls need critical thinking skills as well as changes in their enabling environment^^[[1]](#footnote-1)^^.

There is also evidence that economic assets, in addition to having value on their own, have benefits in other areas of women and girls’ lives. For example, girls who have fewer economic assets are more likely to have exchanged sex for money, gifts or shelter compared to girls with more assets^[[2]](#footnote-2)^; girls who received a cash benefit for schooling showed a decline in early marriage, teenage pregnancy and self-reported sexual activity^[[3]](#footnote-3)^; and adding a financial education component to a life-skills intervention resulted in significantly greater positive impact in changing sexual behaviours.^[[4]](#footnote-4)^ There is also evidence that while health interventions alone can improve health knowledge and attitudes, they are typically not sufficient to achieve health behaviour change. For example, lack of economic assets has been cited as a barrier to translating sexual and reproductive health knowledge into behaviour change, especially in adolescence, as girls are often financially dependent on men and therefore lose decision making power in their sexual relationships.^[[5]](#footnote-5)^ However, economic interventions on their own are not enough, and can even increase risk among adolescents^[[6]](#footnote-6)^ ^[[7]](#footnote-7)^, while programmes that have combined economic strengthening interventions with prevention of violence and health components have had a positive outcome on all three areas^[[8]](#footnote-8)^.

Therefore, the evidence points to multi-sectoral approaches for adolescent girls programming as a promising strategy for achieving high-level impact. With a comprehensive set of social, health, and economic assets built through a package of interventions, girls will be empowered to take more control over their decisions as to sexual partners, financial responsibilities, and health care.

The evidence, however, is lacking on which combination of interventions would be the most impactful. Further, in order to provide policy-relevant evidence that governments, donors, and other stakeholders can use to scale up successful packages for girls, it is critical to understand the costs attached to each package and which level of change can be expected for each financial input.

The main objectives of the research will be to determine: 1) Which combination(s) of interventions produce(s) the greatest change in the wellbeing of adolescent girls? and 2) What is the most cost-effective way to achieve the greatest impact for adolescent girls?

A randomized controlled trial design will be used to examine the impact of the intervention packages on various indicators of adolescent fertility and well being of girls over a four-year period (see Appendix A). The project will be implemented in Wajir County and Nairobi slums. Wajir County is located in the mainly rural, semi-arid region of Northern Kenya, and inhabited by a mainly pastoralist, Muslim population of Somali origin. The Nairobi slums represent the urban poor population of Nairobi. They are characterized by high population density, lack of public utilities and services, and high rates of migration.

In Nairobi, girls will be randomized to intervention packages at the individual level. All girls aged 11 to 14 residing within the study site will be listed and 3,000 girls will be selected based on a vulnerability index that excludes girls from high socio-economic status households. These girls will then be randomized to one of the four study arms. In addition, using the same procedure in an external control site, 750 girls will be interviewed but will not directly benefit from the interventions. In Wajir randomization will occur at the cluster level. Clusters will be defined as school-catchment areas of a fixed radius around a public primary school that includes all families sending girls aged 11 to 14 to the given public primary school. Based on power calculations, we will have 80 clusters (20 per arm), which will have approximately 3,200 girls in the given age group).

The intervention packages that will be tested are:

1. Violence Prevention
2. Violence Prevention + Education
3. Violence Prevention + Education + Health
4. Violence Prevention + Education + Health + Wealth Creation

The selection of package combinations was based on three factors: 1) prioritizing what component effects were important to isolate; 2) generating evidence on new interventions and new combinations of interventions for which there is none; and 3) testing packages with varying implementation costs.

The hypothesis is that this diverse asset base, once acquired, will lead to increased educational attainment, delayed childbearing and marriage, fewer unintended pregnancies, less experience of violence, and increased economic assets. Using both quantitative and qualitative data, we will also test the causal mechanisms driving the impact for each of the four intervention sectors, based on the hypotheses shown in Appendix B.

1. **Intervention**

Population Council has been involved in the design of the interventions, but the interventions will be implemented by Save the Children (Wajir) and Plan International (Nairobi). Information on these implementing partners has been provided in Section 3. All interventions will be implemented for a period of two years in research sites (see timeline in Appendix C). The beneficiaries will be girls selected for the research study as per the procedures described in Section 8.

In each sector AGI-K will implement one intervention so that it will be possible to attribute any change to that specific intervention component. In cases where more than one component is proposed, the rationale and evidence will be provided.

1. Violence Prevention

The violence prevention intervention will be a community level intervention using the community dialogues and contracts strategy.

AGI-K will adapt the UNDP/Concern model (described below), integrating promising approaches from the PC Guatemala and East Africa activities and will run this component for the full two years of the AGI-K intervention. It is envisioned that a core committee will be established within the first month. The committee will comprise religious leaders, community leaders, parents, teachers, and young men and women. This group will go through a facilitated process where they learn to identify the key issues in the community that lead to the under-valuing of girls, come up with a “contract” of steps to address those issues, and then carry out activities to implement the contract over the course of the intervention. A small amount of funds will be available for each community for implementation of the agreed upon steps. Careful monitoring will be done of the committee meetings and committee-led activities.

Community Conversations Model

Facilitators will be trained on the Concern International/UNDP-adapted Community Conversations (CC) model. The training will cover the development of facilitation skills required for social transformation, participatory tools needed to create community enlightenment and the classical process for guiding conversations. Each community conversations group will follow a manual that takes them through a structured process.

The manual for the CCs will include a schedule with specific session topics, activities and tools to be used during each session with the CC members. Through these guided sessions, the CC members will build relationships with each other, identify the major concerns related to violence against girls and the value of girls in their communities, explore the root causes of these concerns as well as the resources available to address them, make decisions and an action plan to address the concerns, implement their action plan using the CC fund, and reflect and review on their progress.

Figure 1. Community Conversations Framework

1. Education: Conditional Cash Transfer

The education intervention will be a conditional cash transfer based on 80% attendance over the course of a term. Attendance will be defined as attending the whole day of school. Girls who are both in and out of school at the start of the intervention will be eligible. There are four components to the transfer:

1. Fees: if applicable, to be paid at the start of each term, directly to the school, with the amount varying by site. The payment of first term fees in each year will be conditional upon enrolment. The payment of second and third term fees will be conditional upon attaining 80% attendance in the prior term (except for the fees for *first term of the program*, which will be paid conditional on enrolment, even though it will be the second term of the year).
2. Cash Transfer to Head of Household: to be paid twice per term, the first payment based on attendance during the first month of the term and the second payment will be made after the third month based on 80% attendance during the second and third months of the term. Each transfer will be 1750 KES, for a total of 3500 KES per term. This reflects 10% of monthly household consumption in Wajir and 15% for urban slums in Nairobi. This amount was determined based on needs assessments in the study communities, average household consumption in each site, and comparison to other education CCTs amounts^[[9]](#footnote-9)^.
3. Kits: at the start of each term, a girl will receive a schooling kit with the following items: 4 packs of sanitary towels, 2 panties, 1 small container of petroleum jelly, 1 bar of soap, 1 exercisebook, 1 pencil, 1 pen. The kit follows the same conditioning rules as the school fees.
4. School Incentive: In addition to the fees, each school will receive a top-up of 500 KES/ per girl enrolled in the CCT programme. This will be paid together with the fees payments at the start of the term.

Figure 2. AGI-K Education Cash Transfer Payment Schedule

In April 2015, eligible households will register for the cash transfer programme, providing details about the girl, school, and will provided with the payment details. At the time of registration, parents/guardians will also participate in a sensitization session about the importance and value of girls’ education. A household/girl will be eligible for the CCT for the entire two year period, regardless if she met the conditions the term before (e.g. she could receive the transfer during the first period of the term, but not the second if her attendance levels drop).

Cash transfer payments to the household will be made via Equity Bank. In Wajir, the payments will use the same infrastructure as the Hunger Safety Net Programme (HSNP), through which all households in Wajir have completed paperwork for account opening with Equity Bank. In Nairobi, the payments will use the Orphans and Vulnerable Children infrastructure – a cash transfer programme through which recipients have accounts with Equity Bank that operate special wallets to receive and transact with the transfer. Households that already have Equity accounts can have the payment wallet added to those accounts. Those that do not will be facilitated to open Equity accounts at the time of registration into the education CCT.

1. Health

The health intervention will follow the Council’s safe spaces model^[[10]](#footnote-10)^ in which girls will meet in groups once a week under the guidance of a female mentor from that community. Groups will be segmented by age (10-12 v. 13-14) in Nairobi and by schooling status in Wajir. The groups will follow a structured curriculum, but also allow time for open discussion. The curriculum will include topics on hygiene, nutrition, HIV/AIDS, sexual and reproductive health, communication/negotiation skills, gender norms, sexual and gender based violence, female genital mutilation/cutting (FGM/C), early marriage, leadership skills and relationships^[[11]](#footnote-11)^. The curriculum will be adapted from existing modules, used by the Council in similar interventions, for each geographical setting (Wajir/Nairobi slums) to ensure that the context is culturally-appropriate and acceptable by the community. In addition, certain sessions will be marked for the 13-14 year olds only if determined to be topics appropriate only for the older cohort. The information from the needs assessment, as well as previous partner experience working in these sites, will guide the decisions on which sessions are acceptable.

Groups will meet in spaces identified in the community as safe and appropriate for girls, which could be community halls, schools, churches, mosques, community leaders’ residences, and others. The groups will meet over the course of two years. This dose is in line with other similar programmes using the safe spaces model, as while one purpose is to deliver content, the other is to help girls build social assets such as friendships and adult mentor relationships. These relationships take time to build and past experience has shown that 18-24 months is the typical dose necessary for these effects to take root.

1. Wealth Creation

The wealth creation intervention will be composed of savings and financial education, layered on to the safe spaces groups. A financial education (FE) curriculum will be added to the group sessions for the girls in the wealth creation arm. These sessions will cover basic money management skills such as saving, budgeting, and differentiating between needs and wants. There are currently several curricula developed by Population Council and past partners for this age group, and they will need only minor adaptations for the urban site, with cultural adaptations for the northern site.

In the Wajir, girls will receive a homebank (piggybank) after the first unit of five to six financial education sessions. The homebank will have 300 Kenyan shillings (KES) in cash in it. The purpose of the cash is to allow girls to immediately put into effect the skills learned through the financial education sessions. The results of two recent literature reviews of evaluations of financial education suggest that financial education is most effective in achieving sustained behaviour change when the lessons learned can be put into practice at the time of the education^[[12]](#footnote-12)^ ^[[13]](#footnote-13)^. Therefore, the design includes an immediate opportunity for girls to start acting on savings goals and budgets. An additional 300 KES will be distributed at the start of the second year of the intervention for the same purpose.

In Nairobi, girls will be facilitated to open Smata Accounts with Kenya Postbank. These accounts, initially developed as part of the Youthsave programme^[[14]](#footnote-14)^, are unique from other “junior accounts” in the market in that these are accounts that are opened and managed by the girls themselves. Girls will need a co-signatory to open and make withdrawals from the account. The co-signatory will be a trusted adult above the age of 18 and selected by the girl. The programme will cover the 200 KES minimum operating balance, as well as make a 300 KES deposit at account opening, as well as at the start of the second year following the same reasoning listed above.

1. **Study Goals/Objectives**

The overall goal of the project is to reduce adolescent fertility in the long-run by improving the wellbeing of girls in programme sites.

The main objectives of this research will be to determine:

1. Which combination(s) of interventions produce(s) the greatest change in the wellbeing of adolescent girls?
2. What is the most cost-effective way to achieve the greatest impact for adolescent girls?

This IRB protocol requests approval of the overall AGI-K research design including activities to be implemented in 2016 and beyond. Specific forms and instruments for the household listing, school mapping, baseline survey components and monitoring data are submitted in full and we ask for approval to implement these activities immediately. In order to fully describe the AGI-K project, we are also including descriptions of the procedures for the midterm survey, endline survey, biomarker data collection, and qualitative survey but will submit amendments including the informed consent forms and instruments for these activities at a later date.

1. **Overview Table:**

|  | **Data Gathering Activities** | | | |
| --- | --- | --- | --- | --- |
| *Note: these are just examples and do not represent all possible study activities* | HH listing | School Mapping | Baseline Survey | Midterm Survey |
| **Study population** | Adult member in the household, all households in study sites in Nairobi | Administrators at all public primary schools in study sites | Girls aged 11 to 14 and adult member in household in all study sites | Girls aged 11-14 and adult member interviewed at baseline |
| **Sample size** | Approximately 60,000 households. | Approximately 400 schools | 3,200 girls & 3,200 parents/guardians in Wajir  3,750 girls & 3,750 parents/guardians in Nairobi | 2,150 girls & 2,150 parents/guardians in Wajir  3,093 girls & 3,093 parents/guardians in Nairobi |
| **Location of activity** | Household | School | Household | Household |
| **Timing** | October – December, 2014 | October – December, 2014 | January – March, 2015 | April – October, 2017 |
| **Method** | Short form | Observation & short form | Interview | Interview |
| **Informed Consent document (Appendix #)** | HH listing consent, Annex 1 |  | Parental permission form, and adolescent assent form, Annex 1 | To be submitted in amendment |
| **Study Instrument (Appendix #)** | HH listing form, Annex 2 | School mapping form, Annex 3 | Girls baseline survey, Annex 4 | To be submitted in amendment |

|  | **Data Gathering Activities (Continued)** | | | |
| --- | --- | --- | --- | --- |
| *Note: these are just examples and do not represent all possible study activities* | Endline Survey | Biomarker Data | Monitoring Data | Qualitative Study |
| **Study population** | Girls aged 11-14 and adult member interviewed at baseline | Girls participating in midterm and endline surveys ages 15 and above | Girls participating in the study and community dialogue members. | Girls selected from baseline sample, community dialogue members and key stakeholders |
| **Sample size** | 2,150 girls & 2,150 parents/guardians in Wajir  3,093 girls & 3,093 parents/guardians in Nairobi | 2,394 girls in Nairobi | 3,200 girls in Wajir  3,750 girls in Nairobi  Approximately 1,900 adults | In-depth interviews: 56 girls & 70 community members at each round |
| **Location of activity** | Household | Household | Household , school, program sites, financial institutions | Household |
| **Timing** | April – October, 2019 | April – June, 2017  April – June, 2019 | May 2015 – April 2017 | R1: May – August 2016 & R2: April – May, 2018 |
| **Method** | Interview | Finger-prick blood tests | Mobile phone apps, biometrics, and program documents | In-depth interviews |
| **Informed Consent document (Appendix #)** | To be submitted in amendment | To be submitted in amendment |  | Annex 8 |
| **Study Instrument (Appendix #)** | To be submitted in amendment | To be submitted in amendment |  | Annex 9 |

**8. Data Gathering Activity 1: Household Listing**

**a. Subject Population**

- In Nairobi urban slums, an initial household listing will take place in the study sites (TBD) with the assistance of maps obtained from KNBS and local village elders. The research team will screen and list all members of households within the selected sub-locations that have an adolescent girl between the ages of 10 and 15 (approximately 60,000 to 90,000 households). The respondents will be adults (age 18+) residing in each household. The household listing will allow us to identify eligible adolescent girls between the ages of 11 and 14 in each household. The listing form will also collect basic information on household characteristics that will be used to rate households using a vulnerability index.
- In Wajir County, data from the national Hunger Safety Net Program (HSNP), a conditional cash transfer program for vulnerable households in the arid and semi-arid lands of Kenya, will be used to provide the sampling frame. These data are from a program registration of all households in Northeastern Kenya in 2013. The Council has obtained a subset of the data for approximately 66,714 households with a girl between the ages of 7 and 14 in Wajir County. The data include information on heads of households, individuals’ ages, education attainment, health and household characteristics as well as village-level GPS coordinates. The Council has received permission from the National Drought Management Authority, the owners of the data, to use the data for the purpose described here.

**b. Research Protocol**

- The household listing will be conducted between October and December, 2014 in the study site in Nairobi slums. Maps of the structures in each enumeration area within the study site will be obtained from the Kenya National Bureau of Statistics prior to the activity. Trained research assistants will visit every household, identify an adult who can answer questions about members of the household, and interview them using a short form (see Annex 2) to collect the required information. They will begin by asking several screener questions. If there is at least one girl between the ages of 10 and 15 residing in the household, they will complete the household roster and the brief background questionnaire on household characteristics. Interviewers will make up to three visits to each household if there is no one present.

**c. Risks and Benefits to Subjects**

- There is a minimal risk of breach of confidentiality, since the household listing form includes contact information and identifies the location of the household. However, no sensitive information will be collected as part of the listing activity.
- The benefit of participating in the listing is that if there is a girl residing in the household, between the ages of 11 and 14, she will be eligible to participate in the study and may receive direct benefit from the interventions.

**d. Steps to Minimize Risks**

- To minimize the risk of loss of privacy, data will be collected using electronic data capture (tablets) and will be stored on password-protected computers at the Population Council office.

**e. Confidentiality**

- All data that includes identifying information will be stored on password-protected computers that will only be accessible by authorized research staff.
- Records will be kept throughout the life of the study and will be kept for up to 10 years afterwards to allow researchers to follow-up with the girls for future survey rounds if external funding is obtained.
- The information collected in the household listing activity will be used to evaluate characteristics of eligible households whose girls chose to participate in the interventions, and those that did not. This information will be provided at aggregate and no identifying information will be used.

**f. Compensation**

- The participants will not be paid for their participation in the household listing activity.

**g. Informed Consent Process**

- Verbal informed consent will be obtained at the time of the household listing. Interviewers will approach the household and read a brief statement (see Annex 1) explaining the purpose of the household listing, assuring respondents that participation is completely voluntary and that it is their right to refuse to answer any questions or stop the interview at any point. Written informed consent will not be collected for the household listing.
- Before the study commences, permission for the study will be obtained nationally by obtaining a research permit from the National Council of Science and Technology in Kenya. We will also obtain local IRB approval from the AMREF Ethical & Scientific Review Committee. Study coordinators will also make courtesy calls to local government officials, including the District/Sub-County Commissioner, the District/Sub-County Officer, and the chiefs in the selected sub-locations. The study team will work with village elders to identify areas in the community.

**8. Data Gathering Activity 2: School Mapping**

**a. Subject Population**

- The study population is all public primary schools in Wajir County, all primary schools in Nairobi County where program girls are attending, and secondary schools in both counties where program girls enrol during the study period.

**b. Research Protocol**

- Prior to the start of the baseline survey, the Council will engage an independent consultant to conduct a mapping exercise to locate all public primary schools in Wajir County and map them using GPS coordinates. The Ministry of Education, Science and Technology will be engaged to provide support for the exercise in exchange for receiving access to the data. While conducting the mapping, a school quality survey (see Annex 3) will be completed that collects basic indicators on a school – including but not limited to teacher-student ratio, public v. private, number of female teachers, and the presence/type of latrines. These quality measures will later be accounted for in the analysis as a mediating factor in the relationship between the intervention and schooling outcomes. The survey will include observation of school facilities and questions about the school that will be answered by the school administrator.
- The school mapping activity will take place in October, 2014 in Wajir and in May, 2015 in Nairobi and will be conducted by an independent consultant.
- In Nairobi, once girls are invited to participate in the study, a list of the schools they are attending will be generated and the school quality survey will be completed on each school.
- In both Nairobi and Wajir, a school quality survey will also be completed for any secondary schools that the girls enrol in during the two-year study period.

**c. Risks and Benefits to Subjects**

- There is no risk to the school or to administrators in providing this information. The questions will be standardized to those collected by the Ministry of Education, Science and Technology for their Education Management Information System, which will be publicly available.
- The benefit of participating in the exercise is that the schools in eligible study sites will benefit directly if the catchment area is randomized to receive the education intervention. The Ministry of Education in Wajir County will benefit from having a map of public primary schools, and information on the quality of these schools.

**d. Steps to Minimize Risks**

- N/A

**e. Confidentiality**

- Basic information on schools will be made available to the Ministry of Education, Science and Technology and may be made publicly available or distributed to other interested parties.
- The school quality information will be linked to survey data and no identifying information will be used in papers or conferences.

**f. Compensation**

- No compensation will be provided.

**g. Informed Consent Process**

- This activity involves gathering basic indicators for schools in the region. No personal information about individuals will be collected, but data on number of students and the profile of the faculty will be reported in aggregate.  As such we are not seeking informed consent. However, we will ask for permission from a school administrator to gather this information after providing him/her with information about the purpose of the study.

**8. Data Collection Activity 3: Baseline Survey of Adolescent Girls**

**a. Subject Population**

- The study population is girls between the ages of 11 to 14 residing in Nairobi slums or in school-catchment areas in Wajir. The participants for the baseline survey will be 3,200 girls between aged 11 to 14 in Wajir and 3,750 girls in the same age group in Nairobi (3,000 girls in the intervention sites and 750 girls in an external control site). In addition, in each household, the parent/guardian who provides consent for the girl to participate in the survey will also participate in a short interview on the household characteristics (3,200 parents/guardians in Wajir and 3,750 parents/guardians in Nairobi).
- For the Nairobi study, a household listing will be conducted to identify all girls in the target age group. The listing form will collect basic household-level data that will be used to rate households using a vulnerability index. We will select an indicator that provides adequate variation and is able to identify girls who are most vulnerable. Possible indicators include having a head of household with no education, or being behind in schooling vis-à-vis one’s age. Based on the selected vulnerability index, households of high socio-economic status (SES) will be excluded and all other households with an eligible girl between the ages of 11 and 14 will be eligible for the study.

For the Wajir study, school-catchment areas will be identified through a school-mapping activity. All catchment areas in study sites will be eligible for the project (approximately 80 total). Within these catchment areas, data from the HSNP will be used to provide the sampling frame. All girls between the ages of 11 and 14 during the time of the survey, residing in school-catchment areas within study sites will be listed from this data and households will be randomly selected for the baseline interview.

- All eligible girls who consent to the interview will be interviewed at baseline. Once the survey is complete, clusters (Wajir) and households with eligible girls (Nairobi) will be randomly assigned to study arms during a public lottery. In Wajir, there is a risk of unequal distribution of clans across study arms, despite randomization. Information on clans will be collected during the school mapping exercise that can be used to stratify by clans, if necessary. Once randomization is completed, girls will be invited to participate in the various components of the programmes, based on their assigned study arms. Mentors and other programme staff will visit the girl in her home and discuss the benefits of the programme with both the girl and her mentor. They will then invite the girl to participate in the programme, obtain the necessary consents and follow-up with the girl to ensure that she has enrolled in programme activities.

**b. Research Protocol**

- The baseline survey will be conducted between January and March, 2015, before intervention activities take place. This period was chosen to avoid the holiday months of December and April, when families are likely to travel upcountry or to visit relatives. This also avoids the rainy seasons in Wajir of October through December and April through June.
- Trained interviewers will be grouped into teams of 8-10, each team with a supervisor to provide directly quality assurance at the time and location of interviewing. The data manager and study coordinator will be in the field during data collection, reviewing the quality of the surveys on a daily basis, addressing trouble areas, and sending the data to the investigators on a daily basis for review. Once respondents have been randomly selected, interviewers will visit households to conduct the interview with selected respondents. If the respondent is not present at the household on the day of the interviewer visit, the interviewer will pay at least three visits to the household in order to locate and interview the selected respondent, similar to the procedure used in the Demographic and Health Surveys (DHS). No replacement will be undertaken, so as not to bias the sample toward ‘stay-at-home’ respondents. Before the surveys are conducted, informed consent will be obtained from parents/guardians of girls, and from girls themselves. Parental consent will not be required from emancipated minors (e.g. married girls) and girls of the age of 18 or older (during the endline survey). To the extent possible, interviews will be conducted in a private area, with visual and auditory privacy.
- Survey instruments (see Annex 4) will be implemented by electronic data capture: Computer-Assisted Personal-Interviewing (CAPI) on tablet computers. CAPI is a process of data capture in which the interviewer reads the question from a computer screen and enters the participant’s response directly into a handheld or tablet device.
- Due to the longitudinal nature of the study, it is important to ask all girls basic information on experience of marriage, female circumcision, violence and sexual intercourse. This data will provide the baseline for examining changes in the program indicators over time (see Appendix D). Since many of the respondents are unlikely to have experienced these events at the study age (11 to 14), they will be skipped out of answering additional questions once they indicate that they have never experienced each event. Some sections on sexual behaviour, intimate-partner violence and family planning will only be asked of those who have been married or have had sex, particularly for Wajir where sex generally occurs within the context of marriage.

**c. Risks and Benefits to Subjects**

RISKS

- Primary ethical concerns of this study include a) that participation in the study may put the respondent at risk of increased violence or psychological trauma related to recounting violent or traumatic experiences, and b) that this study includes minors, below the age of 18. WHO has produced ethical guidelines related to interviewing respondents on gender based violence; Population Council and FHI have produced ethical guidelines related to interviewing children and adolescents. Both volumes will be drawn upon to ensure that the most ethically sound research is conducted that protects the respondents and minimized harm, including psychological trauma. In addition, interviewer training will include sessions on research ethics and protection of subjects. All questions asked in the survey have been previously asked with no adverse effects in other Council studies with adolescent girls.
- This study includes sensitive questions that might possibly cause adolescents to experience stress or anxiety. However, the elements of this survey have been used in other settings with girls of similar ages and circumstances with no evidence of distress. The information obtained through these questions is critical for understanding the relationship between economic assets and sexual exploitation. This study will make significant contributions to scientific knowledge and provide guidance for program managers and policy-makers as they design interventions to improve the lives of adolescent girls by promoting education, preventing violence and building their social, economic and health assets.
- This study will include minors below the age of 18. In accordance with the UN Convention on the Rights of the Child (1989), which has been ratified by Kenya, a minor is defined as a person who has not reached the age of 18. As children, these participants are vulnerable and unable to make decisions about study participation without the consent of a parent or guardian. Generally, there are no more than minimal psychological and health risks for the subject participating in the study. We will obtain signed permission from parents or guardians of all minors.
- Due to the fact that we will interview the same respondents at baseline, midterm and endline, personal identifying information will collected and maintained from participants. While this information will be stored separately from the completed questionnaires, there is a minor risk of breach of confidentiality.

BENEFITS

- Respondents will receive no compensation for their participation in the study. Participants in this control sites will have the opportunity to experience mainly indirect benefits from the study, in the sense that the information they are providing will directly inform the development of the interventions to prevent violence improve health, increase economic assets and promote education. Participants in Wajir and in intervention sites in Nairobi will directly benefit through the interventions that will be implemented in their communities or that they will participate directly based on their assigned study arm.
- Our experience in previous youth studies in the East African region suggests that many youth benefit from the research experience through sharing their experiences with others and being given the opportunity to express themselves. Youth respondents from prior studies in Ethiopia, Kenya, Zimbabwe, and South Africa often describe that people have rarely shown an interest in them or asked their experiences and opinions prior to the interview experience. Respondents may benefit from the experience of being given a platform to express themselves and recount their ideas and experiences. Results from their participation will also be shared with government, private, and NGO organizations, which will inform future programs and improve nuanced and context appropriate programs for adolescent girls.

**d. Steps to Minimize Risks**

- To minimize the risk of adolescents not being able to provide informed consent, we will also obtain consent from a parent/guardian in addition to the girl’s assent Girls will only be allowed to participate in the survey if their parents agree and they also assent.
- To minimize the risk of psychological trauma, all girls who report traumatic events will be referred to local partner organizations that have gender-based violence response services, including psycho-social counseling, medical services and legal services.
- To minimize the risk of loss of privacy, data will be collected using electronic data capture (tablets) and will be stored on password-protected computers at the Population Council office.

**e. Confidentiality**

- All interviews will be conducted in private, in a physical space in which other study participants cannot hear the questions or participant responses.
- All data collected as part of this project will be kept under lock and key at the Population Council offices in Nairobi, and stored in a secure facility. All electronic data will be stored on password-protected computers and back up files will be encrypted and stored on secure media. Computers, filing cabinets and related research equipment/facilities that could reveal identifying information will be only accessible to authorized research staff. The consent forms, along with identification information, will be kept separately so that names cannot be linked to the behavioral information collected during the study. Computer printouts and related documents will be disposed of properly. Research assistants and other study personnel will be educated on the importance of confidentiality. All reasonable efforts to ensure confidentiality is not breached will be made.
- Records will be kept throughout the life of the study and will be kept for up to 10 years afterwards to allow researchers to follow-up with the girls for future survey rounds if external funding is obtained.
- No identifying information will be included in any reports or papers.
- Data collected in this study may be deposited and made publicly available to other researchers through the Population Council’s Girl Innovation, Research, and Learning (GIRL) Center Adolescent Data Hub. The study team will de-identify data, including all names, addresses, and indirect identifiers, before depositing it in the Adolescent Data Hub. GPS coordinates will not be included in deposited data. The GIRL Center will conduct additional checks to confirm that all identifiable data are removed. If identifiable information are found, the GIRL Center will either remove or anonymize the identifiable data. The GIRL Center’s procedures for de-identifying data are attached (Annex 11).

**f. Compensation**

- The participants will not be paid for their participation in the baseline survey.

**g. Informed Consent Process**

- Before the study commences, permission for the study will be obtained nationally by obtaining a research permit from the National Council of Science and Technology in Kenya. We will also seek local IRB approval from the AMREF Ethical & Scientific Review Committee. Study coordinators will also make courtesy calls to local government officials, including the District/Sub-County Commissioner, the District/Sub-County Officer, and the chiefs in the selected sub-locations. The study team will work with village elders to identify areas in the community.
- Written informed consent will be obtained prior to all data collection activities by the interviewer in a private setting. Following established informed consent protocols, each respondent will be provided a thorough explanation of the purpose of the study, the privacy and confidentiality of their responses, and the process and extent of participation. The consent form will be read aloud by interviewers to the respondents, whereupon the respondent will be asked if they agree to participate. Before the interviewer records the consent, the respondent will be asked if they have any questions or concerns about the study, which will be recorded by the interviewer.
- As part of the Population Council’s monitoring program, the subject will be asked to acknowledge the possibility that an interview may be requested by a representative of the Population Council to determine if informed consent occurred. If an interview is requested, the subject will have the option of accepting or declining the interview.
- If the girl is under 18 years of age, written informed consent from a parent or guardian will be obtained prior to the interview. Written assent from the girls will also be obtained separately for the interview. For those 18 and older and for married girls, who are considered to be emancipated minors, informed consent for the interview will be obtained only from the girl.
- All face-to-face interviews (FTFI) will take place in a physical space in which other study participants cannot hear the questions or participant responses.

**8. Data Collection Activity 4: Midterm Survey for Adolescent Girls**

**a. Subject Population**

- The sample will include all girls who participated in the baseline survey (2, 394 Kibera, 699 Huruma = 3, 093 in Nairobi and 2, 150 in Wajir). Girls will be located and asked to participate in a second interview.

**b. Research Protocol**

- The midterm survey will be conducted between April and October, 2017.
- The procedures and locations are the same as for the Baseline Survey described above. In addition, meetings will be held with parents of girls in the control arms to sensitize them about the research activity beforehand, to reduce the number of refusals.

**c. Risks and Benefits to Subjects**

- The risks and benefits include those described in the Baseline Survey section above. In addition, participants will directly benefit by receiving a small reimbursement for participation in the survey in the amount of KES 200 (USD $2). The reimbursement will be provided to girls even if they choose not to complete the entire survey. This benefit has been added due to the ongoing engagement of study participants in the longitudinal study and the need to further engage them in tracking surveys and the endline survey that will be conducted in 2019.

**d. Steps to Minimize Risks**

- The steps to minimize risks are the same as for the Baseline Survey described above.

**e. Confidentiality**

- The steps to ensure confidentiality are the same as for the Baseline Survey described above.

**f. Compensation**

- The participants will receive a small reimbursement for participation in the survey, in the amount of KES 200 (USD $2).

**g. Informed Consent Process**

- The informed consent procedures are the same as for the Baseline Survey described above.

**8. Data Collection Activity 5: Endline Survey for Adolescent Girls**

**a. Subject Population**

- The sample will include all girls who participated in the baseline survey (2, 394 Kibera, 699 Huruma = 3, 093 in Nairobi and 2, 150 in Wajir). Girls will be located and asked to participate in a third interview.

**b. Research Protocol**

- The endline survey will be conducted between April and October, 2019.
- The procedures and locations are the same as for the Baseline Survey described above.

**c. Risks and Benefits to Subjects**

- The risks and benefits include those described in the Baseline Survey section above. In addition, participants will directly benefit by receiving a small reimbursement for participation in the survey in the amount of KES 200 (USD $2). The reimbursement will be provided to girls even if they choose not to complete the entire survey.

**d. Steps to Minimize Risks**

- The steps to minimize risks are the same as for the Baseline Survey described above.

**e. Confidentiality**

- The steps to ensure confidentiality are the same as for the Baseline Survey described above.

**f. Compensation**

- The participants will receive a small reimbursement for participation in the survey, in the amount of KES 200 (USD $2).

**g. Informed Consent Process**

- The informed consent procedures are the same as for the Baseline Survey described above.

**8. Data Collection Activity 6: Biomarker Data**

**a. Subject Population**

- The study population is 2, 394 girls aged 15 or older residing in Kibera (Nairobi), who participated in the Baseline Survey. At midline we anticipate this to be approximately half of the sample and at endline the full sample will be eligible.

**b. Research Protocol**

- Biomarker data on HIV and HSV-2 will be collected at midterm and endline for all girls ages 15 and older.
- All HIV, HSV-2 specimens will be collected at the household at the time of the midterm or endline survey or where the adolescent feels most comfortable. Interviewers will receive training on finger-prick blood collection, and will be certified in HIV Testing and Services based on Ministry of Health guidelines. The study will also make available a private space in the community to conduct the interview and testing in cases where confidential interviewing and testing cannot be done at the home. HIV test results will be provided directly to the adolescent girl at time of specimen collection. If the adolescent is uncomfortable receiving her results at the household she can obtain them at the local health clinic as per procedures discussed below. All HSV-2 test results will be provided only at the local health clinic. Details of the testing and counseling procedures for HIV and HSV-2 are as follows:HIV: HIV status will be determined via capillary blood draws obtained from finger pricks. HIV tests will be conducted by trained and certified voluntary counseling and testing (VCT) staff. In accordance with Ministry of Health guidelines, we will conduct serial testing using Determine™ and First Response™. Both tests have a very high sensitivity (100%) and specificity (>99%) in controlled clinic evaluations, including a controlled laboratory setting in rural Kenya (Foglia et al. 2004). The first test used will be Determine™. If the test is reactive, the counsellor will proceed with a second test using First Response™. If the second test is also reactive, the participant will be confirmed positive. If the second test is negative, a third test, will be used as a tiebreaker.^^[[15]](#footnote-15)^^  If the third test is non-reactive, the participant will be confirmed negative, if reactive, confirmed positive

After the HIV test is complete (approximately 15 minutes), the respondent will be provided the test results if she wishes. Post-test counselling will be conducted by a trained and certified VCT counsellor and, if applicable, the respondent will be provided referrals for follow-up testing and care (for positive results). Post-test counselling will be guided by National Guidelines for HIV Testing and Counselling in Kenya (Annex 5). If the respondent does not wish to receive her results at the household or if confidentiality cannot be maintained, the respondent will be informed that she can receive her results at participating health centres. Respondents will be provided vouchers with identification numbers to receive their test results. The results will be available at the health center for a minimum of one month after the completion of fieldwork for each round.

- HSV-2: The HSV-2 biological specimens will be collected via finger prick. A sample of whole blood will be collected and stored in microtainers. To conduct the HSV-2 laboratory test, serum will be derived from the whole blood. The HSV-2 specimen will be tested at a partner laboratory using the Kalon ELISA antibody test, for which the sensitivity and specificity have been found to be high (100%) in clinical evaluations when compared to Western Blot. Indeterminate results will not be retested since their prevalence is so low as to make it impractical. After specimen collection all participants will be provided information about HSV-2 detection, symptoms, safe sex practices and treatment options. Respondents will be provided vouchers with identification numbers to receive their test results at pre-agreed upon health centers. The results will be available at the health center for a minimum of 1 month after the completion of fieldwork. A validation of the laboratory testing procedures will be conducted prior to testing of specimens for the main study to assure the quality of laboratory testing protocols.

**c. Risks and Benefits to Subjects**

RISKS

- There is a possibility that a psychological risk (worry/anxiety) may exist regarding the respondent’s learning of his/her anaemia, HIV and HSV status. To address this risk, adolescents will be provided VCT services, including counselling about their status, safe sex, healthy living and treatment options for STIs. National protocols and guidelines for VCT will be followed and interviewers will be certified as Ministry of Health HIV VCT counselors.
- Although the potential is low, there are risks regarding involuntary disclosure of HIV or HSV status or other information provided to interviewers. The risks include embarrassment and stigmatization within the community.
- There are minor health risks from the biological specimen collection. For the capillary blood finger prick, the only potential health risk is infection in the punctured area if standard procedures are not followed. However, with proper training regarding sterilization procedures, this risk is considered to be very low. If more serious side effects or if an infection should occur, the participant will be referred to the appropriate medical personnel for treatment at no cost. Contact information for medical personnel or location for treatment will be provided.

BENEFITS

- Participants will directly benefit from the availability of HIV and HSV-2 VCT, as well as from the availability of counselling for preventing STI transmission and safe sex. Such information has the potential for protecting study participants from STI acquisition, as well as potentially protecting their sexual partners. STI positive participants will be informed about treatment options and provided with appropriate referrals.
- Given the availability of anti-retroviral therapy in Kenya, knowledge of HIV positive status will lead to better health outcomes in the long term if ARV treatment is sought by the participant. Although treatment of HSV-2 is not common in Kenya, knowledge of infection status may lead to behaviors that protect sexual partners and improve outcomes for women who are pregnant and know their status. Knowledge of anaemia status might lead to treatment and improvement in nutrition that improves general health status for girls.

**d. Steps to Minimize Risks**

- To minimize psychological risks, adolescents will be provided HTS services, including counselling about their status, safe sex, healthy living and treatment options for STIs. National protocols and guidelines for HTS will be followed and interviewers will be certified as Ministry of Health HTS counsellors.
- To minimize risk of breach of confidentiality, all biological specimens and test results will be recorded separately from any personal identifying information and will contain a participant ID code. The file that linking the participant ID codes to their survey responses or other identifying information will be stored in a password protected computer and available only to study investigators.

**e. Confidentiality**

- See above, regarding minimizing risk to breach of confidentiality. Additional steps to ensure confidentiality are the same as those described in the Baseline Survey.

**f. Compensation**

- The participants will not be paid for the collection of biomarker data.

**g. Informed Consent Process**

- Once parents/guardians have consented to the mid-term or endline interviews, they will be taken through a separate consenting process for the collection of biomarker data from their girls. Parents/guardians will be informed that the interviewer will not disclose the test results to the parents/guardians, regardless of the results. If they agree, the girl will be taken through the informed consent process for the interview as described for the Baseline Survey, above. After completing the face-to-face interview, the girls will be taken through the consenting process for the biomarker data collection. Girls will be told that they may refuse to obtain HIV results at the household, that they may obtain anaemia, HIV and HSV-2 results at centralized sites at a later time or they may refuse to obtain their results altogether. They will also be told that they can withdraw from the study at any time without retribution. Consent will be documented with a signature and date.

**8. Data Collection Activity 7: Qualitative Study**

**a. Subject Population**

- The study population is girls who participated in the Baseline Survey and participants of community dialogues (violence prevention intervention), and key stakeholders. In the second round, a total of 56 girls (24 in Nairobi and 32 in Wajir) will be purposively selected to participate in in-depth interviews. At least 70 (30 Nairobi, 40 Wajir) in-depth interviews will be conducted with community dialogue members and community stakeholders (local government officials, religious leaders, teachers, school heads, mentors, community conversation facilitators, parents of adolescent girls, etc) in each of the two qualitative data collection rounds.
- Adolescent girls will be purposively sampled from the quantitative respondents for in-depth interviews and stratified by demographic and other characteristics that could influence study outcomes. For community dialogue members and all focus group participants, purposive sampling will be used to recruit participants based on pre-identified target profiles in collaboration with implementing partners and local leadership.

**b. Research Protocol**

- The first round will be conducted between May and August, 2016, one year into the intervention implementation. The second round will be conducted between April and May, 2018, one year after the end of the interventions. The study sample will be different, but the samples will be selected using the same method as in the first round. The timing of the two qualitative data collection periods are meant to complement the quantitative study.
- Once participants are identified, trained interviewers will visit them in their homes, review the consent forms and conduct interviews and focus groups in a private area, with visual and auditory privacy. Interviewers will follow moderator’s guides and in-depth interview guides that will provide the main questions and possible probes to elicit in-depth responses. Focus group discussions will take place in public places, such as churches or schools, where there is adequate privacy. For in-depth interviews, each respondent may be visited over two to three rounds of interview, progressively covering more sensitive topics on successful visits. This technique is valuable in promoting candour during the interview process, particularly on sensitive topics. In particular, respondents, at times, change their description of experiences on the third visit, for example describing experiences of violence and/or rape, whereas none was reported in earlier sessions.
- All interviews and focus group discussions will be tape recorded and transcribed verbatim. The data analysis of the transcribed data will consist of multiple and iterative readings of the resulting transcripts as well as coding of emergent themes using ATLAS.ti or a similar qualitative analysis software. Qualitative findings will complement and contextualize findings from quantitative survey through triangulation and mixed method approaches.

**c. Risks and Benefits to Subjects**

RISKS

- The risks are the same as those described above for the Baseline Survey, including the risk of increased trauma related to recounting violent or traumatic experiences and the participation of minors.
- There is an additional risk of loss to privacy due to the tape-recording of interviews and focus groups.

BENEFITS

- Adolescent respondents will receive KES 200 compensation for their participation in the study. Adult in-depth interview respondents will receive compensation in the amount of KES 300. In addition to this, adult participants will receive a transport reimbursement. Participants in the first round of the study will continue to benefit from the interventions being implemented in their communities. All participants will benefit from the research experience through sharing experiences with others and being given the opportunity to express themselves by providing information that will be used to guide future programs.

**d. Steps to Minimize Risks**

- To minimize the risk of adolescents not being able to provide informed consent, we will also obtain consent from a parent/guardian.
- To minimize the risk of psychological trauma, all girls who report traumatic events will be referred to local partner organizations that have gender-based violence response services, including psycho-social counseling, medical services and legal services.
- To minimize the risk of loss of privacy, respondents will be asked not to state names or give other identifying information on the recordings. The digital recordings will be stored on password-protected computers at the Population Council office, and will be identified using a serial number. They will not contain any identifying information.

**e. Confidentiality**

- Steps to ensure confidentiality are the same as those described for the Baseline Survey, above.
- Recordings will be destroyed at the end of the study period.
- No identifying information will be included in any reports or papers.

**f. Compensation**

- Participants will receive a small compensation for their participation in the qualitative study. Adolescent respondents will receive KES 200 compensation for their participation in the study. Adult in-depth interview respondents will receive compensation in the amount of KES 300. In addition to this, adult focus group participants will receive a transport reimbursement for their travel to the venue where the meeting will take place.

**g. Informed Consent Process**

- The informed consent process is the same as that described for the Baseline Survey above. Consent from parents/guardians and participants will be obtained prior to conducting in-depth interviews and focus groups. Consent forms will inform the respondents that the interviews will be recorded, and that the information will be transcribed, stored and analyzed without any identifying information.

**8. Data Collection Activity 8: Monitoring Data**

**a. Subject Population**

- The study population is all girls who participate in the Baseline Survey and are randomized to the three arms that include direct program participation by girls. (2,250 Nairobi, 2,400 Wajir) and all adults (approximately 1,900 adults) who participate in the community dialogues (violence prevention intervention). Excluded are girls in the violence prevention only arm, who will not be directly participating in program activities and girls in the external control site in Nairobi.

**b. Research Protocol**

- Monitoring data will be collected throughout the intervention period.
- Regular monitoring data is critical for two main reasons: 1) using attendance data in real time is a critical programme management tool that allows for quality control of programme implementation and programme staff can identify areas that have low attendance, are off track in the programme timeline, etc. and make adjustments quickly; and 2) attendance data can be merged into the longitudinal quantitative data described above through a unique serial number for each programme participant – this will allow for a dose-response analysis which will provide information on the relationship between level of exposure to a programme and level of impact. Consequently, these data will both facilitate programme implementation and supplement the quantitative analysis.
- For safe spaces groups, girls’ attendance and topics covered during group meetings will be tracked by program mentors using a mobile application linked to a web-based platform. Field staff will input attendance and participation data on mobile phones and upload it to the system. Site coordinators, implementing partners and the research team will be able to examine the progress of the implementation using these data as well as visually view graphs of attendance per girl, group, community, intervention package, among other monitoring outcomes. This database will highlight problem areas much faster than using attendance registers filled in by hand that need to then be transported to an office, entered into a system, and then analysed.
- Mentors will be assessed on their knowledge of the training topics, values and facilitation skills. This will be done using pre and post-test tools and periodic structured observations of their facilitation skills during group meetings. Questions on the girls’ interactions and relationships with their mentors will be added to the surveys at the mid-term evaluation.
- Information on community dialogues participation will also be tracked by facilitators using a mobile app like the one described above. This will include attendance, topics covered, and objectives achieved.
- Cash transfer payments will be monitored by tracking the distribution and receipt of payments by project beneficiaries. Project staff will also keep track of the distribution of education kits to girls, and the amounts and receipt of school incentives by participating schools and the amounts of school fees paid directly to the school for each girl.
- Data on school attendance will be collected using biometrics, due to the inaccuracy of school attendance registers. Girls will be required to place a finger on a biometric reader at the school once in the morning and again in the afternoon. The data will be transferred by program staff into a database, and will be used to establish girls’ eligibility for the education cash transfers and kits during the next disbursement period. Field staff will also make copies of the school registers and conduct spot checks by visiting schools unannounced and taking attendance. This information will be used to validate the school attendance records.
- For Nairobi, for girls in the fourth study arm that includes an intervention on wealth creation, account transaction data (deposits and withdrawals) will be collected directly from the financial institution. Girls’ AGI ID (assigned at the time of the baseline survey) will be entered into the bank system during account opening and will be used to link survey data to monitoring data, while maintaining the participants’ anonymity.
- All girls who participate in the Baseline Survey will be tracked to monitor their enrolment and attendance status, and to keep updated information on their whereabouts for future survey waves. This is because the analysis will examine outcomes for all girls randomized to a given arm (Intent-to-treat analysis), rather than only looking at girls who chose to participate. On a quarterly basis, research assistants will conduct phone calls to households of all girls who are actively participating in the programme to update their contact information in the study database. For all other interviewed girls, including those who did not enrol in the programme, those who may have dropped out, girls in the violence-prevention arm and girls in the control site, research assistants will first call, then visit their homes and track their whereabouts once a quarter. Programme staff and mentors will follow-up with girls who may have dropped out of the programme to find out their reasons for dropping out and address any issues that they can to encourage them to continue their participation in the programme.

**c. Risks and Benefits to Subjects**

RISKS

- The collection of monitoring data will not increase risk for girls because their anonymity will be maintained using a unique ID, and no identifying information will be included in data provided by the financial institution.
- There is a minor risk of loss of privacy due to the collection of contact information for tracking purposes.

BENEFITS

- Respondents will receive no compensation for their participation in the study. Participants in the control sites will have the opportunity to experience mainly indirect benefits from the study, in the sense that the information they are providing will directly inform the development of the interventions to prevent violence improve health, increase economic assets and promote education. Participants in Wajir and in intervention sites in Nairobi will directly benefit through the interventions that will be implemented in their communities or that they will participate directly based on their assigned study arm.

**d. Steps to Minimize Risks**

- To minimize the risk of loss of privacy, data will be collected using biometrics and mobile phones or electronic data capture and will be stored on password-protected computers at the Population Council office. In addition, no identifying information will be included in the data provided by the financial institution.

**e. Confidentiality**

- Steps to ensure confidentiality of monitoring data are the same as those described for the Baseline Survey, above.

**f. Compensation**

- The participants will not be paid for the collection of monitoring data.

**g. Informed Consent Process**

- After the completion of the baseline survey and randomization to study arms, girls and their parents/guardians will be invited to participate in the program and informed about the intervention activities and the types of monitoring data that will be collected as part of the intervention. Both parents/guardians and girls will be asked to sign consent/assent forms for program participation, that includes collection of monitoring data as described above. A separate form will be developed for each arm to describe the interventions/monitoring activities unique to each arm (i.e. education attendance, group attendance and account transactions).

**9. Data Management**

Survey instruments will be implemented by electronic data capture using Computer-Assisted Personal-Interviewing (CAPI) on tablet computers. The Study Coordinator and data manager will check all completed questionnaires for completeness and accuracy. Data will be exported into Stata for further internal consistency checks and analysis.

Electronic data will be collected and stored on secure tablet computers with multiple layers of security, requiring login at the computer and application level. The data will be backed-up daily on external media, encrypted and stored in a secure environment. No personal identifying information will be included with the participant’s survey responses. The file that link participants to their responses will be stored in a password protected computer and available only to study investigators and coordinators.

All qualitative interviews and focus group discussions will be tape recorded with the permission of the participants. No personal identifying information other than the participant IDs will be included on the recordings. All taped conversations will be transcribed in the language of the interview and the transcripts will then be translated into English; only the participant’s ID will be included on the transcripts. A separate electronic file (Microsoft Excel) will be used to link the quantitative and qualitative data. Security and backup procedures for the electronic data produced for the qualitative data will follow the same protocols as discussed above.

**10. Training and Qualifications of Personnel**

Quantitative female survey interviewers with at least a diploma or Bachelor’s degree will be recruited from each of the project locations. Supervisors with considerably more research experience will be recruited to oversee interviewers. Qualitative interviewers will have a university degree (preferably Masters Degrees in Anthropology and Sociology) and have previous experience conducting qualitative research.

For the Nairobi sample, the study investigators will make use of an existing pool of highly experienced and trained quantitative and qualitative data collectors, all of whom have already had experience interviewing young adolescent girls as part of the Council’s past studies. For the Wajir sample, the Council will make use of the female data collectors trained by Save the Children and/or the HSNP, as they are already experienced conducting interviews and entering data electronically on tablets. Interviewers will be familiar with the study locations and the local culture, as well as the local language. Recruitment from within regions will provide additional benefits for residents of that region, as well as development opportunities for young people from the area.

All interviewers will attend a ten-day training on the details of the survey instruments. Separate trainings will be conducted for the Wajir and Nairobi studies due to the different survey languages and in order to reduce travel costs of interviewers. Training will include an item by item review of the research instruments, mock interviews and a practice interview done outside the study area. Interviewers will be grouped into teams of 8-10, each team with a supervisor to provide directly quality assurance at the time and location of interviewing. Supervisors will attend the training as well as an additional one-day training devoted to supervision, data quality, and troubleshooting issues in the field. Finally, the data manager and study coordinator will be in the field during data collection, reviewing the quality of the surveys on a daily basis, addressing trouble areas, and sending the data to the investigators on a daily basis for review.

**11. Instrument Development**

The instruments were developed by the study investigators based on previous surveys, study outcomes and indicators to be tested and gaps in the literature. The household listing tool was developed based on previous tools used in Kenya and Zambia in studies of adolescent girls. The school quality tool is based on the Malawi School Quality survey and the Kenya Ministry of Education, Science and Technology school census tool for their Education Management and Information System. The quantitative survey for girls will cover topics such as: household socio-demographic characteristics; schooling history; education attainment: social assets and networks, self-efficacy, locus-of-control, financial literacy, savings and livelihoods, marital and child-bearing aspirations, birth history, experience of physical and sexual harassment and violence, attitudes on FGM, self-reported health and nutrition; reproductive health knowledge, HIV and AIDS risk perception; comprehension in local language and English; excerpts from official mathematics assessments multiple standards (grades); and cognitive testing. The midterm survey will include additional questions to measure exposure to interventions. The household background survey was developed using questions from the Kenya Demographic and Health survey on household assets and living conditions. Other questions were added from previous tools used in the Kenyan context. The purpose of this tool is to collect background information that the girls might not be privy to. The tool also includes some questions on gender norms that will be used to assess changes attributable to the community-level intervention over time. The surveys will be translated into Swahili and Somali. The surveys will be pilot-tested prior to the start of data collection and revised based on feedback from interviewers before data collection begins.

Qualitative data will be collected to provide a better understanding of the causal mechanisms and pathways identified using the quantitative analysis. It is expected to provide an in-depth explanation of what worked and why. Through this data, we will also learn how each intervention directly or indirectly impacted the individual girl as well as the household members and community at large. Furthermore, the data will highlight the strengths and weaknesses of the intervention implementation. Qualitative instruments will be developed by the study investigators in collaboration with the implementing partners. They will be pre-tested in communities within the study regions that were not selected as study sites and revised based on feedback before interviewers begin data collection. To ensure that the research is culturally sensitive, interviewers at both sites will speak the local languages and will be familiar with the local culture. Partners will engage the community before the program starts to inform them about the project, the study objectives, and to involve them in the randomization process through a public lottery. External Advisory Committees will be formed locally in both Nairobi and in Wajir to guide the project and ensure that all cultural issues are identified and addressed.

**12. Annexes**

**Annex 1: Baseline Survey Informed Consent Documents**

**Annex 2: Household Listing Form**

**Annex 3: School Mapping Tool**

**Annex 4: Baseline Survey**

**Annex 5: Kenya VCT Guidelines**

**Annex 6: Midterm Survey Informed Consent Documents**

**Annex 7: Midterm Survey**

**Annex 8: Qualitative Survey Informed Consent Documents**

**Annex 9: Qualitative Survey In-Depth-Interview Guides**

**Annex 10: Endline Survey Informed Consent Documents**

**Annex 11:** **GIRL Center procedures for de-identifying data**

**Ethical Training Certificates**

Included for Karen Austrian, Eunice Muthengi, Paul Hewett and Erica Soler-Hampejsek

**IRB Checklist**

Reviewed

**APPENDIX A: AGI-K RANDOMIZED CONTROL TRIAL STUDY DESIGN**

**APPENDIX B. Causal Mechanisms by Which Interventions Might Affect Timing of Childbearing**

Arm 1

Arm 2

Arm 4

Arm 3

Endline

Endline

Endline

Midterm

Midterm

Midterm

WAJIR:

RANDOM ASSIGNMENT 80 Clusters (~3,200 girls)

NAIROBI:

RANDOM ASSIGNMENT 3,000 GIRLS

Baseline

Baseline

Baseline

Baseline

Midterm

Endline

Baseline

Midterm

Endline

NAIROBI

EXTERNAL CONTROL

SRH Knowledge & Social skills

Savings & Financial Education

Community Dialogues

Conditional Cash Transfer

Improved norms on timing of marriage

Reduced non-consensual sex

Increased enrollment & education attainment

Reduced risks (sex/marriage) driven by economic necessity

Reduced unprotected sex

Improved self-efficacy & decision-making

Causal Mechanism

Programme

Outcome

Legend Causal pathway in Wajir

Causal pathway in Nairobi

**APPENDIX C: AGI-K Timeline**

| **Timeline:** |  |  |
| --- | --- | --- |
|  | **JANUARY 2014** | Start of Inception Phase |
|  | **OCTOBER 2014** | Start of Implementation Phase |
|  | **JANUARY - MARCH 2015** | Baseline Survey data collection |
|  | **APRIL - JULY 2015** | Start of intervention |
|  | **APRIL 2016** | Midterm Implementation Report |
|  | **JULY - AUGUST 2016** | Qualitative Data collection |
|  | **MARCH - JUNE 2017** | End of intervention |
|  | **APRIL - OCTOBER 2017** | Midterm Survey data collection |
|  | **MARCH 2018** | Midterm evaluation report |
|  | **APRIL - MAY 2018** | Qualitative Data collection |
|  | **APRIL – OCTOBER 2019** | Endline Survey data collection |
|  | **MAY 2020** | Final evaluation report |

**APPENDIX D: AGI-K KEY INDICATORS**

| INTERVENTION | INDICATOR 1 | INDICATOR 2 | INDICATOR 3 |
| --- | --- | --- | --- |
| OVERALL | Increased age at first birth | Increased age at first sex | Increased age at marriage |
| VIOLENCE | Decreased experience of gender-based violence | Improved gender norms related to violence |  |
| EDUCATION | Increased mean grade of schooling | Increased rate of primary school completion |  |
| HEALTH | Increased knowledge on sexual and reproductive health | Improved decision-making skills |  |
| WEALTH | Increased knowledge on financial education | Increased saving | Increased participation in income generating activities |

1. Lloyd, C. 2013. “Education for Girls: Alternative Pathways to Girls Empowerment.” A Paper Commissioned by Girl Hub, a strategic partnership between Nike Foundation and the UK Department for International Development. [↑](#footnote-ref-1)
2. Hallman, K. Gendered socioeconomic conditions and HIV risk behaviours among young people in South Africa. 2005. *African Journal of AIDS Research.* 4(1): 37–50. [↑](#footnote-ref-2)
3. Baird, S. et. al. 2010. The short-term impacts of a schooling conditional cash transfer program on the sexual behaviour of young women. *Health Economics.* 19: 55–68. [↑](#footnote-ref-3)
4. Amin, S. et al. 2010. “Enhancing Adolescent Financial Capabilities through Financial Education in Bangladesh.” Population Council. [↑](#footnote-ref-4)
5. Austrian, K. and Anderson, A. Forthcoming. “Barriers and Facilitators to Health Behaviour Change and Economic Activity Among Slum-Dwelling Adolescent Girls in Nairobi, Kenya: The Role of Social, Health and Economic Assets.” [↑](#footnote-ref-5)
6. Dunbar, M., Maternowska, M.C., Kang MS, Laver SM, Mudekunye-Mahaka I, Padian NS. 2009. Findings from SHAZ!: a feasibility study of a microcredit and life-skills HIV prevention intervention to reduce risk among adolescent female orphans in Zimbabwe. Journal of Prevention and Intervention in the Community, Apr 38(2):147-61. [↑](#footnote-ref-6)
7. Austrian, K. and Muthengi, E. 2013. “Safe and Smart Savings Products for Vulnerable Adolescent Girls in Kenya and Uganda: Evaluation Report.” New York: Population Council. [↑](#footnote-ref-7)
8. Pronyk PM, et al. 2006. “Effect of a structural intervention for the prevention of intimate partner violence and HIV in rural South Africa: a cluster randomized trial.” *Lancet.* 368: 1973–1983. [↑](#footnote-ref-8)
9. For example, the Zomba education CCT was the equivalent of 15% of household consumption. The Save the Children education CCT study in Garissa is currently 3000/ per month, or 37% of household consumption. [↑](#footnote-ref-9)
10. Austrian and Ghati. 2010. *Girl-Centered Program Design: A Toolkit to Design, Strengthen and Expand Adolescent Girls Programs*. Population Council. [↑](#footnote-ref-10)
11. One aim of the FGM sessions will be to change the attitudes of girls around the practice to affect their continuing the practice with their own daughters. As it is likely that close to 100% of the girls will have already undergone FGM by age 11, the aim is not to change the prevalence of FGM among the study population. [↑](#footnote-ref-11)
12. Miller, M., Reichelstein, J., Salas, C., and Zia, B. 2014. *Can You Help Someone Become Financially Capable?*

    *A Meta-Analysis of the Literature*. Policy Research Working Paper 6745. World Bank. [↑](#footnote-ref-12)
13. Fernandes, D., Lynch, J., and Netemeyer, R. Forthcoming. “Financial Literacy, Financial Education and Downstream Financial Behaviours.” *Management Science*. [↑](#footnote-ref-13)
14. www.youthsave.org [↑](#footnote-ref-14)
15. Formerly, SD Bioline HIV 1/2 3.0 was used as a tiebreaker for indeterminate cases in Kenya. However, recently the Kenyan Ministry of Health (MOH) has discontinued Bioline’s use due to quality assurance issues. If a third test has not been designated by the MOH by study start date, serum collected as part of the HSV-2 will be ELISA tested at the laboratory as the tiebreaker. For the small number of cases for which this would occur, the participant would be revisited to receive her test results and counselling. [↑](#footnote-ref-15)
