# Supplementary material for: Impacts of multisectoral cash plus programs after four years in an urban informal settlement: Adolescent Girls Initiative-Kenya (AGI-K) randomized trial
Source: PLoS One. 2022 Feb 7;17(2):e0262858. doi: 10.1371/journal.pone.0262858 (PMC8820646; doi:10.1371/journal.pone.0262858)
Supplement: S3 File — (DOCX) [file pone.0262858.s012.docx]

Link to publicly available questionnaire: <https://www.popcouncil.org/uploads/pdfs/2021SBSR_AGI-K_EndlineSurveyInstruments.xlsx>

Link to publicly available interview guides: <https://www.popcouncil.org/uploads/pdfs/2021SBSR_AGI-K_MidlineQualInterviewGuides.pdf>
