# Supplementary material for: Impacts of multisectoral cash plus programs after four years in an urban informal settlement: Adolescent Girls Initiative-Kenya (AGI-K) randomized trial
Source: PLoS One. 2022 Feb 7;17(2):e0262858. doi: 10.1371/journal.pone.0262858 (PMC8820646; doi:10.1371/journal.pone.0262858)
Supplement: Attachment — Submitted filename: renamed_567d5.docx [file pone.0262858.s013.docx]

**COMMENTS – Manuscript PONE-D-21-06054**

Title: “Impact of a cash transfer and girls empowerment program on early sexual debut and fertility in a Kenyan urban informal settlement: results from a mixed-methods randomized trial”

**Important note: This review pertains only to ‘statistical aspects’ of the study and so** **‘clinical aspects’ [like medical importance, relevance of the study,** **‘clinical significance and implication(s)’ of the whole study, etc.] are to be evaluated [should be assessed] separately/independently. Further please note that any ‘statistical review’ is generally done under the assumption that (such) study specific methodological [as well as execution] issues are perfectly taken care of by the investigator(s). This review is not an exception to that and so does not cover clinical aspects {however, seldom comments are made only if those issues are intimately / scientifically related & intermingle with ‘statistical aspects’ of the study}. Agreed that ‘statistical methods’ are used as just tools here, however, they are vital part of methodology [and so should be given due importance].**

**COMMENTS:** In my opinion the title should have been just “impact of a multisectoral program for women Empowerment” because [in ‘Methods’ section of ‘abstract’ it is clarified by saying ‘The interventions included community dialogues on the value of girls (violence prevention), a conditional cash transfer (education), weekly group meetings with health and life skills training (health), and financial literacy training and savings activities (wealth).’ which means that] “cash transfer” is just one component of women empowerment ‘program’. Therefore, the question is “Why inclusion of ‘**a cash transfer**’ in title?”. {‘Conclusion’ section of ‘abstract’ has no mention of “cash transfer” component of this ‘program’}. Similarly, mention of ‘**early sexual debut and fertility**’ in title is questionable. Moreover, please explain what exactly you intend to indicate by term ‘Mixed-Methods Randomized Trial’ in the title?

Further, I am doubtful about whether the used one can be classified as ‘a randomized trial design’ [not sure if just random allocation means you have used a randomized trial design]. I am not worried about the ‘label’, however, concerned about the ‘level of evidence’. When you say ‘Post-hoc analysis was carried out on girls who were 13-15-years-old at baseline’, do you mean that you performed ‘sub-group’ analysis on this group? Please clarify what is ‘Post-hoc analysis’. Part of the conclusion [The need to implement such interventions in early adolescence, before negative outcomes crystallize may mean that a long follow up period is necessary to be able to observe the full impact of the intervention as the girls’ transition into young adulthood] is not from this study, it may be the authors’ opinion formed from this experience. I wonder if the investigator(s) are allowed to include in conclusion lesson(s) learned from the study?

I feel that overall, though the study has potential, the ‘presentation’ is not ‘precise’ [I mean exact or to-the-point], particularly the ‘Background’ section. On page 10 (first sentence) is ‘A random sample of eligible girls was selected for the baseline survey’ which is appearing after ‘random allocation’. Is this random sample different than earlier one? How correct it is? What exactly ‘you are trying convey’? by this. Remember that this is a scientific/academic document and so all details should be clearly/correctly communicated. In the same paragraph, further you say ‘The baseline behavioral survey was conducted after public randomization and the start of the intervention.’, what do you mean by that? What do you mean by ‘the censored nature of the indicators’?

Many {such} confusing statements [example: For girls 13 years and older at baseline, we modelled HSV-2 prevalence in 2019 and incidence between 2017 and 2019 for the sample of girls testing negative in 2017] are found throughout the article (them may be correct, however, confusing for readers). Re-drafting of the complete manuscript is necessary, in my opinion. Information given regarding ‘Sample Size Calculations’ is not very clear or convincing. I guess, the Adolescent Girls Initiative-Kenya (AGI-K) is a different study (not made clear anywhere), if so, why the power of this study is quoted from that study protocol (reference 31, The quantitative study was powered to detect differences in the prevalence of first birth and number of grades attained between the V-only and each of the other three arms at endline, four years after the start of the intervention when girls in the sample would be 15–19 years old)? From the statement made that ‘The objective of this study is to assesses the program impacts on the primary outcome of delayed childbearing, as well as on a range of secondary outcomes, two years after its completion, when the girls were 15–19 years old’, it seems that this one is a program evaluation study [because if different than (AGI-K) study, will have a separate ‘power’]. Is not it the investigator’s responsibility to make that clear? Sample size per se of this study is alright (large enough), but the argument is not convincing.

Why ‘Power analysis’ was conducted for a two-sample proportions test when the study had four arms? Please refer to ‘Randomization’ section. What do mean while saying ‘High population density in urban Kibera meant it was possible to reach a large number of girls with excludable interventions, making an individual-level randomized design feasible’? Please make clear. Clarify whether AGI-K is the program name or is it (an independent) study?

In my considered opinion, there is no point in identifying / enumerating / highlighting such loopholes / confusing statements endlessly. I recommend complete redrafting of the manuscript.
