# Supplementary material for: Impacts of multisectoral cash plus programs after four years in an urban informal settlement: Adolescent Girls Initiative-Kenya (AGI-K) randomized trial
Source: PLoS One. 2022 Feb 7;17(2):e0262858. doi: 10.1371/journal.pone.0262858 (PMC8820646; doi:10.1371/journal.pone.0262858)
Supplement: Attachment — Submitted filename: Author response to reviews for PONE 10thDec2021.docx [file pone.0262858.s015.docx]

PONE-D-21-06054
Impacts of multisectoral cash plus programs after four years in an urban informal settlement: Adolescent Girls Initiative-Kenya (AGI-K) randomized trial
PLOS ONE

Dear Dr. Oldenburg,

We want to sincerely thank you and the reviewers for providing further comments. We have taken time to review these comments and have made the necessary changes. We believe that the changes made have strengthened the manuscript and hope that we have managed to address all the concerns sufficiently. All the changes are saved as track changes. In our resubmission we include the following documents:

- This rebuttal letter that responds to each point raised by the academic editor and reviewer(s).
- A marked-up copy of our manuscript that highlights changes made to the original version labeled 'Revised Manuscript with Track Changes'.
- An unmarked version of our revised paper without tracked changes labeled 'Manuscript'.

Yours sincerely,

Dr. Beth Kangwana

**Author response to second round of reviews for PONE-D-21-06054R1**

*We thank the reviewers for the additional valuable comments and have redrafted the paper to address them.*

Please review your reference list to ensure that it is complete and correct. If you have cited papers that have been retracted, please include the rationale for doing so in the manuscript text, or remove these references and replace them with relevant current references. Any changes to the reference list should be mentioned in the rebuttal letter that accompanies your revised manuscript. If you need to cite a retracted article, indicate the article’s retracted status in the References list and also include a citation and full reference for the retraction notice.

*Response: We have reviewed all the references and made some amendments to ensure that they are accurately presented. Please let us know if there are any specific reference you feel need further attention.*

*Due to the changes we have made as a result of suggestions made by the reviewers, we have retracted one reference: Strauss, A. and J. Corbin. 1998. Basics of Qualitative Research: Techniques and Procedures for Developing Grounded Theory. Thousand Oaks, CA: Sage Publications.*

**Reviewer #1**: Although there is some improvement in manuscript after revision, still there are many confusing statements and as said earlier the ‘presentation’ is not ‘precise’, to-the-point. Please check the English language [professional intervention may be needed, I guess]. I have no specific recommendation(s) and think that, ‘let the respected editor decide the future course’.

*Response: After incorporating material and reworking to address Reviewer #2’s comments we 1) reviewed and edited the entire manuscript ourselves and then 2) had it reviewed and edited by the team in the Population Council Inc Knowledge Communications office.*

**Reviewer #2**: Thank you to the authors for their in-depth responses to reviewer comments and for revising this manuscript accordingly. My remaining major recommendations are as follows: (1) the authors speak to transactional sex in the background and prostitution in their findings. As it is written, the authors seem to be overlooking that children and youth cannot consent to sex work. Instead of reporting that girls in their sample engaged in prostitution, I would recommend writing that they have experience child sexual exploitation. If the context and laws in Kenya suggest otherwise, I strongly recommend that the authors highlight this;...

*Response: Thank you for this suggestion. We agree with the reviewer and in line with their suggestion have replaced ‘transactional sex’ in the background and ‘prostitution’ in the findings section with child sexual exploitation.*

...and (2) the section describing qualitative methodology and methods is still insufficient. The authors reported using grounded theory; however, their study is deductive from the Theory of Change and they have not reported nor demonstrated that this study is seeking to develop a theory. Furthermore, information regarding the methods to support a grounded theory methodology is missing.

*Response: Thank you for this comment. We have re-framed the description of the qualitative component as a qualitative evaluation of the trial and clarified the description of the approach to the analysis that was undertaken.*

I would recommend that the authors provide further information regarding data collection (who conducted the interviews and focus groups? How were these conducted?) and data analysis (how was the analysis iterative? How did the authors analyze within and across themes or categories? Did the authors employ axial coding?). I would also suggest that the authors explain how and why the interviews were validated and reviewed for quality assurance. Lastly, I would suggest that the authors provide a qualitative research question and sub-questions (if relevant). Please find further minor recommendations below.

*Response: We have incorporated the additional details regarding the qualitative data collection in the qualitative methodology section. All interviews were conducted by trained interviewers and moderators. The analyses consisted firstly of developing a start-list of codes that were derived from the program’s Theory of Change as well as from the interview guides. Further themes were added as they emerged from reviewing the data. To ensure quality assurance, all transcripts were double coded by two qualified analysts. All double-coded transcripts underwent testing for intercoder agreement and where the Krippendorff’s c-α-binary coefficient was below 0.70, a side-by-side comparison, clarification and reconciliation was carried out on the specific coded transcripts.*

Abstract: Under methods, I would suggest rewording “the value of girls” for the violence prevention outcome.

*Response: Thank you for this suggestion. We have now rephrased “the value of girls” to “unequal gender norms and their consequences” which we feel is a more accurate reflection of what was discussed in the community dialogues.*

Background: Thank you for expanding on the importance of adolescent development. I would suggest reframing that this developmental stage is inherently vulnerable to instead underscore the reasons for vulnerability that you’ve identified in the second sentence of your second paragraph (“lack of economic security, unequal gender norms, pressure from peers to engage in sexual activity, pressure from families to achieve economic security through early marriage, and not living with one’s parents”). I would also recommend rewording or contextualizing the “cascading ramifications of pregnancy” in this section.

*Response: We have modified the first paragraph so that it more clearly describes how developmental changes that occur during adolescence, in combination with certain negative external factors, increase the risk of early pregnancy. We have in addition changed the phrase “cascading ramifications of pregnancy” to “negative potential consequences of pregnancies.”*

Methods: As indicated above, I strongly suggest that the authors revise their qualitative methodology section. In addition, I would recommend that the authors provide an account of the theoretical underpinnings of the Theory of Change that is guiding this study.

*Response: We have made revisions to the qualitative methodology section. In addition, we now reference the theories used to develop the original AGI-K theory of change and provide relevant citations.*

I also wonder if the authors would not consider reframing the qualitative component of this study as a qualitative evaluation of the trial – this reframing would seem to be appropriate given the aims of the qualitative exploration that they have identified.

*Response: Thank you for this suggestion, we agree this is a better characterization of the work. We have made the changes to the ‘Randomization and Data Collection’ and ‘Qualitative Methodology’ sections to indicate that the qualitative component of the study was indeed a qualitative evaluation of the program.*

Ethical considerations: Can the authors please provide an explanation for why parental assent was required for participants between 12-18 years of age?

*Response: Our description was unclear, and we have edited it. Written informed consent was required from all girls 18 years old or older. For girls under 18 years old, written informed consent was required from a parent or guardian, and oral assent from the girl herself.*

Results: I would suggest that the authors begin the results section with an account of their participants’ demographic information and sample sizes. Although the participants provided further context for their qualitative findings, there are few quotes included to support the analysis; this may be due to limited space. I would therefore recommend that the authors include a table of qualitative findings, including themes and participant quotes.

*Response: We have added in a section on participants’ demographic information and sample sizes at the beginning of the qualitative results section. Moreover, we include a table to display the socio-demographic characteristics of the participants that were interviewed for the qualitative evaluation (Table 5). Additional quotes have been incorporated into the qualitative findings section.*

In Table 2, results for a measured cognitive score are presented – can the authors please speak to the purpose of this measure for the study.

*Response: Because an important secondary outcome in the study was education, we measured cognitive scores to enable careful assessment of baseline balance on an important indicator associated with schooling advancement. The cognitive score measure is used as a control in the extended controls models to increase precision of the estimates. The theory of change did not posit that the intervention would strongly influence cognitive scores, so it was measured only at baseline.*

I also wondered why participants in the V-only arm did not receive transfers and ask that the authors briefly provide an explanation for this.

*Response: An objective of the study was to provide rigorous evidence on the causal impacts of providing the three different packages of interventions (all with the education component) against a comparison group. To do so, we used a randomized trial research design in which girls were randomly allocated to four different study arms, including a study arm without the education, health or wealth interventions to be used as the experimental comparison in the quantitative analyses.*

Lastly, I would strongly recommend that the authors revise the language used to explain that girls learned to “protect themselves from boys and against violence”, as this wording currently places the responsibility of experiences of violence on girls.

*Response: Thank you for this suggestion. We have amended the phrase “protect themselves from boys and against violence” to “recognize and protect themselves from sexual and gender-based violence”*
